# Intrinsic neuronal diversity as a substrate for cortical area specialization in primate vision

**DOI:** 10.1038/s41467-026-73734-5

**Published:** 2026-06-09

**Authors:** M. Feyerabend, S. Pommer, S. Mestern, M. S. Jimenez-Sosa, J. Rachel, J. Sunstrum, F. Preuss, V. A. Khatibi, R. Hinkel, M. Mietsch, S. Vijayraghavan, S. Everling, S. Treue, A. F. T. Arnsten, D. A. Lewis, S. J. Tripathy, G. Gonzalez-Burgos, W. Inoue, A. Neef, J. F. Staiger, J. Martinez-Trujillo

**Affiliations:** 1 Department of Physiology and Pharmacology, Schulich School of Medicine and Dentistry, Western University, London, Ontario Canada; 2 Department of Psychiatry, Schulich School of Medicine and Dentistry, Western University, London, Ontario Canada; 3 Department of Clinical Neurological Sciences, Schulich School of Medicine and Dentistry, Western University, London, Ontario Canada; 4 Institute for Neuroanatomy, University Medical Center Göttingen, Georg-August University, Göttingen, Germany; 5 Göttingen Campus Institute for Dynamics of Biological Networks, Göttingen, Germany; 6 Max Planck Institute for Multidisciplinary Sciences, Göttingen, Germany; 7 Laboratory Animal Science Unit, German Primate Center, Leibniz Institute for Primate Research, Göttingen, Germany; 8 German Center for Cardiovascular Research (DZHK), Partner Site Lower Saxony, Göttingen, Germany; 9 Institute for Animal Hygiene, Animal Welfare and Farm Animal Ethology (ITTN), University of Veterinary Medicine Hannover, Hannover, Germany; 10 Cognitive Neuroscience Laboratory, German Primate Center, Leibniz Institute for Primate Research, Göttingen, Germany; 11 Faculty of Biology and Psychology, University of Göttingen, Göttingen, Germany; 12 Department of Neuroscience, Yale Medical School, New Haven, CT USA; 13 Department of Psychiatry, University of Pittsburgh, Pittsburgh, PA USA; 14 Institute of Medical Science, University of Toronto, Toronto, ON Canada; 15 Krembil Centre for Neuroinformatics, Centre for Addiction and Mental Health, Toronto, ON Canada; 16 Department of Psychiatry, University of Toronto, Toronto, ON Canada; 17 Department of Physiology, University of Toronto, Toronto, ON Canada; 18 Max Planck Institute for Dynamics and Self-Organization, Göttingen, Germany; 19 Bernstein Center for Computational Neuroscience, Göttingen, Germany; 20 Institute for Nonlinear Dynamics, Georg-August-University Göttingen, Göttingen, Germany

**Keywords:** Intrinsic excitability, Sensory processing, Extrastriate cortex

## Abstract

In primates, primary visual cortex (V1) circuits support early visual processing, whereas in the lateral prefrontal cortex (LPFC), they support higher-order computations. Here, we characterized the intrinsic electrophysiological (e)-properties and morphology of excitatory (EXC), fast-spiking inhibitory (FSI), and non-fast-spiking inhibitory (NFSI) neurons in layers I-III of V1 and LPFC, of a small primate, the common marmoset. EXC and FSI neurons exhibited broader action potentials in LPFC than in V1. Compared to V1, LPFC EXC neurons exhibited reduced excitability, whereas FSI neurons showed increased excitability. NFSI neurons showed fewer area-specific differences. Notably, EXC and FSI neurons displayed increased bursting in LPFC compared to V1. Morphologically, LPFC-EXC neurons exhibited longer dendritic arbors, whereas LPFC-FSI neurons showed greater axonal complexity relative to V1. Our findings demonstrate area-specific functional and anatomical diversification of major neuronal types in layers I-III of the primate neocortex and provide an open-access resource for studies of neocortical cell types.

## Abstract (web summary)

Primates have a hierarchy of cortical areas that play progressively more complex roles in visual cognition. Here, the authors show that excitatory and inhibitory neurons diversify their intrinsic features across areas to match their functional roles.

## Introduction

Humans and other primates have a highly evolved and hierarchically organized cortical visual processing stream composed of serially connected areas. It starts in the primary visual area V1 and extends far downstream to the lateral prefrontal cortex (LPFC)^1^. Early areas, such as V1, specialize in basic feature detection^2–5^, while far downstream areas, such as LPFC, support working memory, attention, and top-down executive control^6–8^. The differences in neuronal response magnitude, selectivity to different visual stimuli, and involvement in various perceptual and cognitive functions across areas have been extensively documented in the macaque^6,9,10^. This inter-area functional diversity could have different origins, such as variations in expression levels of synaptic receptor subtypes, input-output organization and local circuit connectivity^11–14^, and/or rely on variations in key intrinsic electrophysiological properties (e-features) of single neurons^15,16^. The latter has been difficult to clarify due to the scarcity of comparative studies exploring the e-features of different cell types in distinct areas of the primate neocortex.

Previous studies reported that pyramidal neurons in area V1 and the LPFC of macaques diverge in their morphology^15,17,18^, whereas pyramidal cells in mouse areas V1 and Frontal Cortex (FC) do not show such prominent differences^15^. Some studies in the common marmoset (*Callithrix jacchus*) have reported higher dendritic spine counts in prefrontal cortex pyramidal neurons than in V1^19–26^. One study in macaques also revealed differences in the e-features of pyramidal cells between areas V1 and the LPFC^15^. Interestingly, another study in macaques revealed more subtle differences in the e-features of pyramidal cells in the lateral intraparietal (LIP) area and the LPFC^27^. Such comparative studies of e-features have not yet been extended to other primate species, such as the marmoset. Marmosets are New World monkeys with a lissencephalic cortical organization and a less expanded prefrontal cortex relative to gyrencephalic Old World primates such as macaques; thus, findings obtained in macaques cannot be directly extrapolated to marmosets. Marmosets have been increasingly used as a model for primate vision over the last decade due to their small size, fast reproductive rate, and amenability to circuit manipulations^28–33^. It is therefore essential to document the similarities and differences in neuronal type e-features between marmosets and other primate species, such as macaques and humans.

We have found no previously published comparative studies of neocortical inhibitory interneuron e-features in primates. Inhibitory interneurons form a diverse pool in mammalian brains, powering local computations in various structures, including areas of the cortical visual processing stream^34–41^. Several studies have reported that inhibitory interneurons are composed of diverse and highly evolved cell types in the macaque prefrontal cortex^42–44^, and that the relative proportion of certain types of interneurons varies across cortical areas^45–47^. A recent study reported variations in a subset of morphological features of interneurons between areas V1 and the LPFC in macaques^48^, and at least one study has reported the existence of certain interneuron types with distinctive e-features in the neocortex of humans and macaques but not in rats^49^. Whether and how morphological and e-features of interneurons differ systematically across cortical areas of the visual processing stream (e.g., V1 and LPFC) remains unclear. Addressing this issue has been challenging due to the lack of transgenic markers that enable the identification of cell types during intracellular recordings in primates. Furthermore, studies in large primates, such as macaques, typically have lower sample sizes than those in other species, such as mice^50^. The latter limits the documentation of cell types that occur in small proportions within the sampled region, as well as the statistical power of a study to detect significant differences in variables of interest.

Here, we take advantage of the availability of a small primate, the common marmoset, with a visual system similar to that of macaques and humans, to investigate differences in the e-features of excitatory and inhibitory neurons and their relationship to morphology in areas V1 and LPFC. We use methods, protocols, and tools similar to those the Allen Institute of Brain Science used in the mouse and human cell type databases^51^, i.e., whole-cell patch clamp recordings in acute slices and morphological reconstructions of single neurons. We built a “Primate Cell Type Database” resource (www.primatedatabase.com). It contains 483 characterizations of neuronal e-features across 51 animals, as well as representative morphological reconstructions of neurons mainly in layers I-III of V1 and LPFC. We built a machine learning-based method to classify neurons into three main e-types, Excitatory (EXC), Fast-Spiking Inhibitory (FSI), and Non-Fast-Spiking Inhibitory (NFSI) neurons. We found systematic differences in e-features and morphology between V1 and the LPFC, mainly of EXC and FSI neurons.

## Results

**Fig. 1:**
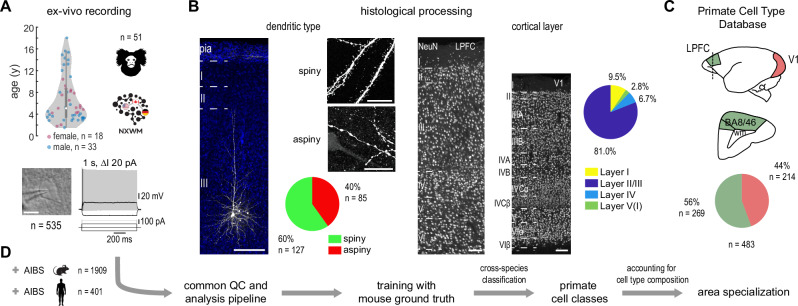
Experimental design and data processing pipeline. **A** Acute slices of LPFC and V1 were obtained from 51 marmosets with a median age of 5.08 years. Boxplot shows median (white circle), IQR (25th–75th percentile, gray box) and maxima/minima (whiskers). Neurons (*n* = 535) were characterized by in vitro electrophysiological (**A**, bottom) and anatomical methods (**B**). Scale bar represents 10 µm. **B** Left image shows a biocytin-filled pyramidal neuron in layer 3 of LPFC; nuclei are stained with DAPI (blue); scale bar equals 150 µm. Small images show example spiny (top) and aspiny (bottom) dendritic morphology; scale bars equal 20 µm. The pie chart shows the distribution of cells with morphologically identified dendritic type. The right images show NeuN staining with layer delineation in LPFC and V1; scale bars equal 100 µm. The right pie-charts represent the distribution of recorded cells by layers for LPFC and V1. Recorded cells were predominantly located in supragranular (I–III) layers. **C** Location for LPFC and V1 in the marmoset brain (top), Brodman areas (BA 8/46) for LPFC in an example coronal section (middle) and pie chart of the proportion of neurons per area (bottom). **D** Processing pipeline of marmoset, mouse and human data. NEURONEX marmoset data were pooled with AIBS mouse and human data to train a cross-species classifier. Source data are provided as a Source Data file.

We recorded from 535 neurons in 51 marmosets. A total of 483 neurons passed quality-control (90.3%), 269 in the LPFC (56%) and 214 in V1 (44%). During the recordings, we targeted cells with apparent (spiny) pyramidal and (aspiny) non-pyramidal (interneuron) morphology, as visualized in supragranular layers I–III of LPFC and V1 slices (Fig. 1A). For cross-species comparison and potential classification, we also used publicly available datasets from the Allen Institute for Brain Science (AIBS), totaling 1,901 mouse cells (primarily acquired from V1) and 401 human cells (acquired from association areas of temporal and frontal lobe). We aligned our data acquisition pipeline with the documented methodology of the respective datasets to minimize the influence of methodological differences (see “Methods”).

**Fig. 2:**
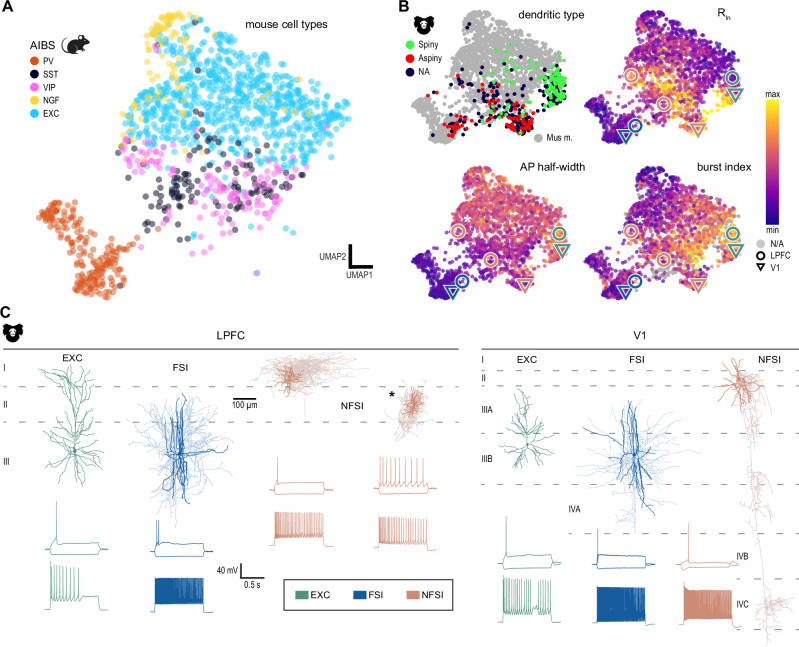
UMAP projection of mouse cell types and key electrophysiological characteristics with example marmoset cells. **A** UMAP plot shows the distribution of selected genetically labeled cell types in the mouse. **B** Distribution of marmoset cells within the whole AIBS mouse dataset, color-coded for dendritic types (top left) and electrophysiological characteristics. Rin: input resistance at steady-state. AP half-width: action potential half-width. Burst index. See supplementary table 1 for more features. Circles (LPFC) and triangles (V1) represent example cells (shown in **C**), with color codes corresponding to the classifier cell types. **C** Example marmoset neuron reconstructions and traces representative of the three cell types. Data were acquired across different laboratories and recording systems. Dendrites are shown in a darker and axons (only FSI and NFSI) in a lighter color shade. Colors reflect the cell type: blue – FSI, ochre – NFSI, green – EXC. LPFC example NFSI neurons are two NGF cells, and the V1 NFSI cell is a vertically-oriented basket cell. Asterisk marks the NGF cell closest to the mouse NGF cluster. Source data are provided as a Source Data file.

We created a multivariate map of electrophysiological features using a Uniform Manifold Approximation and Projection (UMAP) procedure with all available data to examine the distinct profiles of cell types across species (see Fig. 2A, B). The distribution of ground-truth mouse cell type labels suggested that cell identity strongly influenced UMAP topography (Fig. 2A): PV and excitatory (EXC) cells are located at opposite poles of the map. In contrast, VIP and SST cells, which showed a high degree of overlap, are found in between. We next referenced the location of anatomically defined marmoset cell labels. For better clarity, mouse labels were merged into three broader cell classes: fast-spiking interneurons (FSI), non-fast-spiking interneurons (NFSI), and EXC (see “Methods”). Clusters of aspiny and spiny marmoset cells showed a strong correspondence with the cell type gradient observed in the mouse (Fig. 2B). Aspiny cells segregated into two clusters, each in proximity to a different mouse interneuron class (FSI and NFSI). Clusters also showed distinct morphological types that resemble homologous types in the mouse (see Fig. 2C for examples): marmoset (large) basket cells, EXC pyramidal cells and NGF cells. In addition, NFSI showed cells with characteristic morphology, such as vertically-oriented basket cells^42^.

### Classification of marmoset cell types based on intrinsic properties

**Fig. 3:**
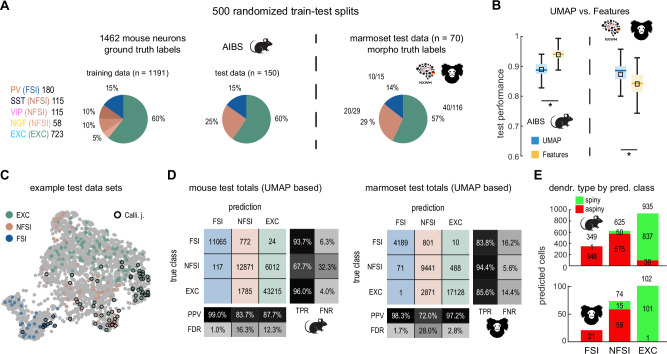
Objective classification of marmoset cells. **A** Pie charts showing cell type composition of training and test data. Three different cell types were used: FSI (blue), NFSI (shades of ocher), merged from SST (yellow), VIP (purple) and NGF (turquoise) cells and EXC (green). Mouse (AIBS) and marmoset test data had a similar proportion of cell types. For marmoset test data, electrophysiology with congruent morphology for FSI and NFSI, respectively, was used respectively. Marmoset EXC cells were drawn from spiny cells. The procedure was repeated 500 times with randomly drawn training and test sets. **B** Performance of classifier for UMAP- or e-feature-approach (29 features) across repetitions. Boxplots show median (colored line), IQR (25th–75th percentile, colored box) and maxima/minima (whiskers). Black rectangles show the mean performance. Median test performance for mouse was 88.7% vs. 94.0% and for marmoset 88.6% vs. 84.3% (UMAP vs. features). Asterisks mark significant differences (Wilcoxon signed-rank test; pmouse = 2.2e-82, pmouse = 2.7e-34). **C** UMAP with example test datasets of mouse (*n* = 150, green, blue and ochre circles) and marmoset (*n* = 70, black bordered circles). **D** Confusion matrices for mouse and marmoset test data showing all classification totals. The positive predictive value (PPV) for FSI and EXC cells is over 90% in the marmoset. FDR = False Discovery Rate, TPR = True Positive Rate, FNR = False Negative Rate. **E**: Bar charts showing the proportion of dendritic type per majority classifier prediction for each dataset used for the classification. Source data are provided as a Source Data file.

We trained a Random Forest classifier with a sample of 1191 mouse neurons to distinguish between FSI, NFSI, and EXC using two separate sets of measurements (Fig. 3A): the raw e-features, and the 29 features entering the UMAP (see Supplementary Table 1). The former produced significantly better test performance in the mouse, while the latter had a significantly better test accuracy in marmosets (signed rank test, 500 train-test splits, 88.6% vs. 84.3%, *p* < 0.0001) (Fig. 3B). Test performance in human data was lower than in marmosets, but the same regardless of whether raw e-features or UMAP features were used (76.5% for both; signed-rank test, *p* = 0.1) (SFig. 4C). In general, classifiers’ performance was stable using either method, and statistical differences were minimal. For the sake of homogeneity, we used the UMAP features for all subsequent predictions.

All marmoset test classifications showed high positive predictive values for FSI and EXC (see Figs. 3D, 98.3% and 97.2%). As expected, the predictive value for NFSI cells was lower (72.0%), because they are a highly diverse group and overlap to a certain extent with FSI and EXC across many e-features. We also validated our predictions against histological data (Fig. 3E): predicted FSI and EXC cells were 100% aspiny and 99.0% spiny, respectively, consistent with anatomical criteria. Furthermore, 20.3% (15 out of 74) of NFS were (sparsely) spiny, which is in close agreement with the classification results (2871 true EXC out of 13113 predicted NFSI, i.e., 21.9%). These findings show that cross-species classification is a reasonable approach for determining three major e-types in our marmoset dataset, EXC, FSI, and NFSI.

### Features of e-types in areas V1 and LPFC

A main purpose of our classification was to identify three classes of e-types and examine possible variability in individual e-features between areas. Since EXC and some NFSI cells can vary significantly across layers, we removed cells from deeper layers ( > III). This resulted in 450 cells for cross-area analysis. From the 29 e-features, we selected the 13 most discriminative e-features (see “**Methods**”, Table 1 and Supplementary Table 1). FSI and EXC cells showed broad divergence across areas. EXC cells significantly differed in 8 different features (R_in_, sag ratio, Rheo, AP half-width, burst index, Adaptation index, Median instantaneous firing rate, and fl-slope). FSI neurons significantly differed in 7 of the same features as EXC cells (R_in_, sag ratio, Rheo, AP half-width, burst index, Adaptation index, Median instantaneous firing rate) and latency. Finally, NFSI neurons significantly differed in 5 features (R_in_, sag ratio, RMP, DFRP90, DFRIQR, see Figure legend for explanation of the variables) (Table 1). The biserial summed absolute rank correlations were 3.51 for FSI and 3.08 for EXC, respectively, indicating substantial aggregate differences across e-features between LPFC and V1 for both cell types. In contrast, NFSI shared only two significant features (R_in_ & sag ratio) with FSI and EXC and showed a summed effect size of 1.11, indicating smaller aggregate cross-area differences.

**Table 1:** Electrophysiological features by cell type and brain area

Feature	LPFC median [25/75%]			V1 median [25/75%]			adj. p		
	EXC	NFSI	FSI	EXC	NFSI	FSI	EXC	NFSI	FSI
R_in_ (MΩ)	205.3 [167.0; 287.5]	467.2 [328.5; 665.4]	243.5 [191.9; 326.9]	463.5 [336.1; 588.3]	576.0 [437.8; 716.2]	184.3 [139.4; 235.0]	**< 0.001**	**0.002**	**0.037**
sag ratio	1.13 [1.09; 1.19]	1.3 [1.16; 1.45]	1.1 [1.08; 1.12]	1.22 [1.15; 1.31]	1.4 [1.2; 1.66]	1.13 [1.11; 1.17]	**< 0.001**	**0.009**	**0.030**
Rheo (pA)	85 [50; 110]	30 [10; 50]	77.5 [70; 110]	25 [10; 35.8]	25 [10; 40]	175 [95; 247.5]	**< 0.001**	0.414	**0.031**
AP half-width (ms)	0.92 [0.8; 1.04]	0.60 [0.52; 0.71]	0.34 [0.30; 0.40]	0.80 [0.71; 0.90]	0.64 [0.54; 0.82]	0.27 [0.24; 0.28]	**< 0.001**	0.105	**0.001**
tau (ms)	27.0 [20.8; 35.8]	21.6 [14.6; 33.4]	13.5 [11.3; 16.1]	27.9 [23.5; 38.8]	24.2 [17.2; 36.5]	12.6 [9.3; 17.2]	0.338	0.338	0.71
burst index	0.74 [0.54; 0.87]	0.48 [0.37; 0.58]	0.43 [0.35; 0.55]	0.64 [0.49; 0.76]	0.46 [0.34; 0.60]	0.33 [0.20; 0.35]	**0.018**	0.809	**0.007**
adap. index	0.775 [0.65; 0.92]	0.5 [0.36; 0.67]	0.46 [0.28; 0.58]	0.67 [0.5; 0.86]	0.51 [0.373; 0.79]	0.585 [0.485; 0.89]	**0.009**	0.142	**0.012**
RMP (mV)	− 72.6 [− 74.9; − 68.3]	− 65.2 [− 66.0; − 64.2]	− 65.8 [− 69.9; − 63.1]	− 72.1 [− 75.4; − 68.5]	− 66.0 [− 68.1; − 64.2]	− 64.8 [− 67.4; 61.8]	0.788	**0.020**	0.624
latency (ms)	102.2 [73.7; 125.6]	63.0 [39.4; 97.9]	14.3 [11.8; 19.8]	90.8 [60.4; 171.4]	63.0 [30.0; 131.8]	14.3 [11.8; 19.8]	0.902	0.902	**0.003**
Median instantaneous firing rate (Hz)	19.5 [15.7; 24.8]	46.1 [31.4; 64.5]	113.0 [99.3; 122.8]	22.3 [19.1; 26.6]	53.0 [38.0; 68.7]	127.6 [112.8; 153.8]	**0.012**	0.075	**0.038**
DFRP90 (Hz)	38.2 [28.5; 50.1]	74.2 [50.8; 99.6]	152.0 [132.6; 169.5]	37.9 [31.2; 55.1]	87.3 [69.7; 140.4]	178.8 [149.0; 197.4]	0.582	**0.002**	0.069
DFRIQR (Hz)	8.4 [6.8; 13.0]	21.5 [16.8; 31.7]	46.8 [38.2; 52.5]	8.8 [6.9; 13.4]	28.6 [22.2; 47.3]	38.2 [35.5; 51.1]	0.451	**< 0.001**	0.451
fI-slope (Hz/pA)	0.06 [0.03; 0.09]	0.40 [0.28; 0.53]	0.62 [0.55; 0.77]	0.12 [0.07; 0.18]	0.31 [0.16; 0.52]	0.70 [0.50; 0.85]	**<0.001**	**0**.080	0.818

Differences were calculated with a two-sided Wilcoxon rank sum test and corrected for multiple comparisons (Benjamini-Hochberg). *P*-values in bold text mark significant differences between brain areas. RMP, resting membrane potential. DFRP90: dynamic frequency range, 90th percentile. DFRIQR: dynamic frequency range, interquartile range. fI-slope: linear fit to f-I curve. Source data are provided as a Source Data file. Exact *p*-Values for significant differences are: pRinEXC = 1.6e-20, psagratioEXC = 9.6e-6, pRheoEXC = 1.1e-20, pAPhalf-widthEXC = 1.5e-5, pDFRIQRNFSI = 9.2e-5, pfislopeEXC = 1.3e-7.

**Fig. 4:**
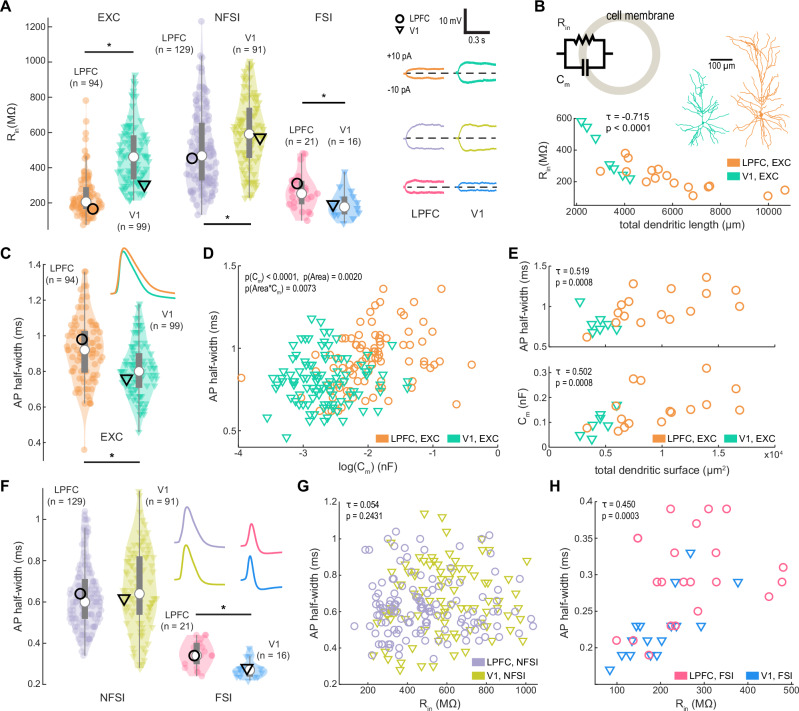
Electrophysiological (e-)type comparison across areas. **A** Violin plots showing Rin by putative cell class and cortical area for EXC (LPFC, orange, V1, turquoise) and FSI (LPFC: pink, V1: blue) and NFSI (LPFC: purple, V1: yellow). Data points (circle: LPFC, triangle: V1) highlighted with black contours indicate cells with example traces to the right. Asterisk mark significant differences (two-tailed Wilcoxon rank sum test; pEXC = 1.6e-20, pNFSI = 0.002, pFSI = 0.037). **B** Scatterplot with total dendritic length and Rin of reconstructed excitatory cells with representative examples for each area (LPFC, orange, V1, turquoise). Correlation was calculated as two-tailed Kendall’s Tau Coefficient. The schematic shows Rin representation. *P* = 1.3e-7. **C** Violin plots of AP half-width of excitatory cells with representative examples of an AP waveform for each area. Asterisk mark significant differences (two-tailed Wilcoxon rank sum test; pEXC = 1.5e-5). **D** Scatterplot of AP half-width and Cm of excitatory cells. Differences are calculated by a linear model with AP half-width as the dependent variable and log Cm and cortical area as independent variables. Coefficient *p*-values are based on two-sided *t* tests without multiple-comparison correction. P(Cm) = 1.1e-5. **E** Scatterplots showing the relationship of total dendritic surface of excitatory cells with AP half-width (top) and Cm (bottom). Correlation is calculated as two-tailed Kendall’s Tau Coefficient. **F** Violin plots showing AP half-width for interneuron cell classes with representative AP waveforms for each class and cortical area. Asterisk mark significant differences (two-tailed Wilcoxon rank sum test; pFSI = 0.001). **G** Scatterplot with Rin and AP half-width of non-fast-spiking interneurons with cortical area in different colors (LPFC: lavender, V1: green). Correlation is calculated as two-tailed Kendall’s Tau Coefficient. **H** Scatterplot with Rin and AP half-width of fast-spiking interneurons with cortical area in different colors (LPFC: pink, V1: blue). Correlation is calculated as two-tailed Kendall’s Tau Coefficient. Violin plots do not show outliers. All box plots show median (white circle), IQR (25th–75th percentile, gray box) and maxima/minima (whiskers). Source data are provided as a Source Data file.

*Passive properties and AP width:* Input resistance (R_in_, the ratio between a change in membrane potential (ΔV) and the amount of injected current) and rheobase (Rheo, the lowest current amplitude that makes a neuron fire) are e-features linked to neuronal excitability. Compared to V1, LPFC EXC cells had a significantly lower R_in_ (205.3 vs. 463.5 MΩ, adj. *p* < 0.001) and higher rheobase (85 pA vs. 25 pA, adj. *p* < 0.001). By contrast, LPFC FSI had significantly higher R_in_ (243.5 vs. 184.3 MΩ, adj. *p* = 0.037) and lower rheobase (77.5 vs. 175 pA, adj. *p* = 0.031) than V1 (Fig. 4A). This inversion of the relationship between EXC and FSI neurons’ excitability from V1 to LPFC suggests that these neuronal types may adjust their e-features to local input strength and the computational role of these areas in vision and cognition (see Discussion). Interestingly, cell size quantified by total dendritic length was inversely correlated with input resistance for EXC (Kendall’s tau, − 0.715, adj. *p* < 0.001, *n* = 23) (Fig. 4B). This relation is to be expected, the overall conductance is simply the product of membrane area and conductance density, although electrotonic conditions will attenuate the contributions of more distal dendritic surface. The fact that similar-sized EXC cells in V1 and LPFC feature similar input resistance suggests that the conductance density is comparable and that the group differences in R_in_ between areas may be linked to total dendritic length^52^, i.e., layers I–III contain smaller and more excitable EXC cells in V1 compared to LPFC.

The action potentials (**APs**) of EXC cells were wider in LPFC than in V1 (Fig. 4C, 0.92 vs. 0.8 ms, adj.* p* < 0.001). We examined how this feature relates to previously established differences in passive properties and morphological features of EXC neurons. Dendritic surface positively correlated with AP half-width (particularly in LPFC, Kendall’s tau, 0.519, adj. *p* < 0.001, *n* = 23, Fig. 4E upper panel). The larger dendritic surface area of EXC cells was also associated with higher membrane capacitance (electrical charge a neuron’s membrane can store per unit voltage), determined by the time constant and R_in_ (Kendall’s tau, 0.502, adj. *p* < 0.001, *n* = 23, Fig. 4E lower panel), indicating that a larger dendritic tree may increase AP width. This influence is presumably mediated by the dendrites’ capacitive load on the soma’s AP-generating conductances (see simulations in ref. ^53^ supplementary material).

To disentangle the effect of (i) cortical area, (ii) membrane capacitance, or (iii) the acquisition system on the diversity of e-features in our sample, we used a linear mixed model with the log-transformed capacitance and cortical area as fixed effects and the individual amplifier system as a random effect (Fig. 4D; see “Methods” for more details). The model accounted for a significant portion of the variance in AP half-width (adj *R*^2^ = 0.304), with an Akaike information criterion (AIC) of − 148.04 and a Bayesian information criterion (BIC) of − 128.56. Both fixed effects were significant for log(C_m_) (β = 0.118 SE = 0.029, t(186) = 4.020 *p* < 0.001) and cortical area (β = − 0.354, SE = 0.111, t(186) = − 3.21, *p* = 0.002), as well as their interaction (*β* = − 0.109, SE = 0.045, t(186) = − 2.431, *p* = 0.0160. The model provides evidence that both the brain area and membrane capacitance correlate with the width of EXC APs. In addition, the increase in AP half-width with membrane capacitance appears to be shallower in V1 (Fig. 4D green triangles) than in LPFC (orange circles).

We also compared FSI and NFSI e-features (Fig. 4F–H). Similar to EXC, FSI in LPFC had significantly broader APs than in V1 (0.34 ms vs. 0.27 ms, adj. p = 0.001), whereas NFSI did not show differences between areas (see Fig. 4F**and** Tab. 1). Interestingly, FSI with high R_in_ had broader APs (Kendall’s tau, 0.450, adj. *p* < 0.001, *n* = 38, Fig. 4H), suggesting that in this cell type, too, subthreshold e-features and AP waveform may be linked and undergo area-specific differentiation. NFSI showed no significant correlation between AP half-width and R_in_. This group showed significantly higher R_in_ in V1 relative to LPFC (Fig. 4A) and a small non-significant trend in the same direction for AP half-width (Fig. 4F–H). These findings indicate a more complex relationship between AP shape and R_in_ in inhibitory interneurons compared to EXC cells, probably caused by the highly diverse cell types that comprise the NFSI group.

Lastly, one e-feature that varies across species and cell types is sag ratio. It quantifies, among other currents, the hyperpolarization-activated I_h_ current (a mixed Na⁺/K⁺ current mediated by HCN channels that regulate membrane potential stability, temporal summation, and the timing of neuronal firing^54^). We compared sag ratio across cell types and found that V1 neurons showed an increase relative to LPFC (LPFC: 1.13, 1.3, 1.1 vs. V1: 1.22, 1.4, 1.13, ranksum test *p* < 0.001, *p* < 0.009, *p* < 0.03, for EXC, NFSI, and FSI, respectively) (see Table 1 and Supplementary Table 1 for more detailed statistics). This result may be linked to the higher mean firing rates we observed in V1 relative to LPFC neurons across cell types (Table 1); i.e., V1 neurons operate over a larger dynamic range than LPFC neurons and therefore may show stronger regulation by HCN channel-mediated I_h_ currents. It may also be linked to the high input resistance we observe in V1 relative to LPFC cells (see Discussion).

### Differences in burst firing and adaptation between V1 and LPFC neurons

**Fig. 5:**
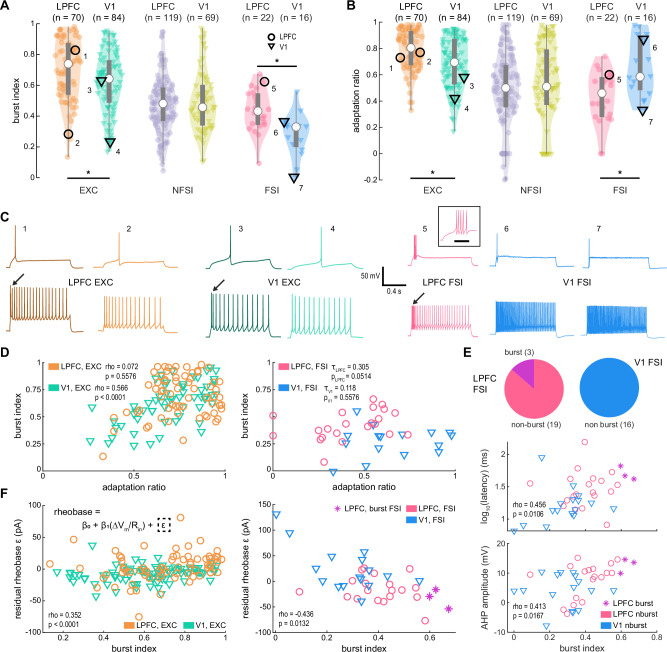
Burst index and adaptation across areas and reference to rheobase. **A**, **B** Violin plots of EXC (LPFC, orange, V1, turquois) and FSI (LPFC: pink, V1: blue) and NFSI (LPFC: purple, V1: yellow) for burst index (**A**) and adaptation ratio (**B**). Numbers mark example bursting (1, 3) and non-bursting (2, 4) EXC cells, as well as bursting (5) and adapting, non-bursting (6, 7) FSI in LPFC/V1. Asterisks mark significant differences (two-tailed Wilcoxon rank sum test; pburstEXC = 0.018, pburstFSI = 0.007, padaptEXC = 0.009, padaptFSI = 0.012). **C** Example of Rheobase and above Rheobase sweeps representative of EXC and FSI e-types. The arrow indicates bursting behavior. Numbers correspond to examples in (**A**, **B**). The inset shows the onset magnification of the Rheobase sweep for the bursting FSI. Scale bar equals 50 ms. **D** Burst index vs. adaptation ratio for EXC (left) and FSI (right). PV1EXC = 1.6e-7. **E** Pie charts show the ratio of bursting cells for FSI neurons and the correlation between latency to first spike, AHP amplitude (y axis), and burst index (x axis). **F** Correlation between model residuals (y axis) and burst index for EXC (left) and FSI neurons (right). PEXC = 2.9e-5. All box plots show median (white circle), IQR (25th–75th percentile, gray box) and maxima/minima (whiskers). Correlations were calculated as two-tailed Spearman’s Rho (**D**–**F**) and Kendall’s Tau Coefficient (**D**). Source data are provided as a Source Data file.

Next, we examined two properties of spike trains that have been shown to shape the activity of neurons in vivo, namely burst firing and adaptation^16^. We quantified bursting using a burst index (see Methods). We found a general trend where EXC neurons showed the largest burst indices, followed by NFSI and FSI neurons, respectively (Fig. 5A). Both EXC and FSI showed significantly higher burst indices in LPFC than in V1 (EXC: 0.740 vs. 0.643, adj. p: 0.0178; FSI: 0.433 vs. 0.330, adj. p: 0.007). NFSI did not show significant differences between areas. To examine the relationship between bursting and adaptation, we also computed the adaptation ratio, which captures changes beyond the first interspike interval (ISI, see Supplementary Table 1 and Fig. 5B). In general, increases in burst firing correlated with increases in adaptation ratio. This was the case for EXC neurons; adaptation ratios in LPFC were higher than in V1. However, this relationship was inverted in FSI neurons; adaptation ratios were significantly higher in V1 than in LPFC (0.46 vs. 0.585, adj. *p* = 0.012). Again, we found no differences between areas for NFSI.

We next examined the correlation between burst index and adaptation ratio by area and cell type (Fig. 5D). For EXC neurons there was no significant correlation in LPFC (Spearman’s rho = 0.072, *p* < 0.5576, *n* = 70) but interestingly, there was a significant correlation in V1 (Spearman’s rho = 0.566, *p* < 0.001, *n* = 84) (Fig. 5D). In FSI the pattern of correlations was inverted between areas (LPFC: Kendall’s tau = 0.305, *p* = 0.0514, *n* = 22, V1: Kendall’s tau = 0.118, *p* = 0.558, *n* = 14).

The significant increase in burst index for LPFC FSI was accompanied by the area-specific occurrence of 3 out of 22 cells responding with bursts of multiple APs at rheobase level (pink traces in Fig. 5C), whereas LPFC EXC neurons fired doublets of action potentials at the beginning of stimulation at medium to high intensities (brown traces). FSI with more prominent burst indices had a higher latency of the first action potential (Spearman’s rho = 0.456, adj. *p* = 0.0106, *n* = 38). They also showed a more depolarized trough at the afterhyperpolarization (Spearman’s rho = 0.413, adj. *p* = 0.0167, *n* = 38).

Furthermore, we examined the relationship between burst index and variables potentially linked to neuronal excitability (Fig. 5F). We first developed linear regression models for each cell type class: Rheo = β0 + β1*(ΔV/R_inHD_) + residuals (ε), where ΔV is the difference between AP threshold and the resting membrane potential, and R_inHD_ is the input resistance. The model explains rheobase by non-ohmic membrane properties plus a residual active contribution. It provides a reasonable goodness-of-fit (EXC: adj. *R*^2^ = 0.774, *p *< 0.001; FSI: adj. *R*^2^ = 0.798, *p *< 0.001). The residuals are the amount of Rheo that is not explained by passive properties. Negative residuals from this model indicate that a part of the depolarization towards threshold was not driven by the pipette current but instead by an intrinsic depolarizing mechanism.

We found a significant negative correlation between rheobase residuals and burst index (Spearman’s rho = − 0.462, *p* < 0.008, see Fig. 5F left) in FSI. The opposite was the case for EXC, where a higher burst index was associated with a weaker but robust positive correlation (Spearman’s rho = 0.352, *p* < 0.001, see Fig. 5F right). In summary, FSI with a higher burst index showed longer latencies to the first spike and smaller afterhyperpolarization amplitudes, consistent with a depolarizing mechanism that activates slowly and persists beyond the first action potential within a burst. Such a mechanism may produce a distinctive class of bursting FSI neurons in LPFC.

### Morphological features of neurons in V1 and LPFC

**Fig. 6:**
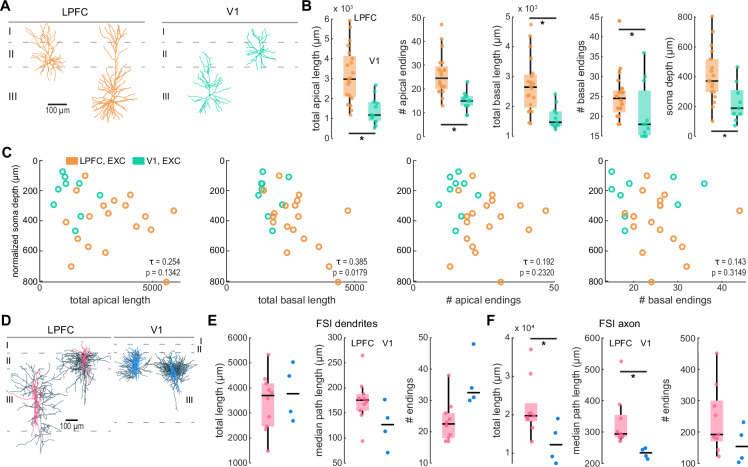
Morphology of EXC and FSI across areas. **A** Examples of reconstructed marmoset EXC neurons for LPFC (left) and V1(right). **B** Distributions of total apical dendritic length, # of apical endings, total dendritic basal length, # basal dendritic endings, and soma depth for EXC neurons in LPFC (orange, *n* = 20) and V1 (turquoise, *n* = 11). Asterisks mark significant differences (two-tailed Wilcoxon rank sum test; ptApiLen = 0.001, p#ApiEnd = 0.001, ptBasLen = 0.002, p#BasEnd = 0.028, pSoma = 0.016). **C** Normalized soma depth of the EXC neurons as a function of total apical dendritic length, total basal length, # apical endings and # basal endings (from left to right). Colors indicate the areas (orange: LPFC, turquoise: V1). Correlations were calculated as two-tailed Kendall’s Tau Coefficient with multiple comparison-corrected *p*-Values (Benjamini-Hochberg). **D** Examples of reconstructed inhibitory neurons in LPFC (left) and V1 (right). The pink and light blue colors indicate the dendrites, and the dark blue colors the axons. **E** Distributions of total dendritic length, median dendritic path length, and # dendritic endings for FSI in LPFC (pink, *n* = 10) and V1 (blue, *n* = 4). **F**: Same variables but for axons. LPFC, *n* = 9; V1, *n* = 4. Asterisks mark significant differences (two-tailed Wilcoxon rank sum test; ptLen = 0.05, pmpLen = 0.028). All box plots show median (black bar), IQR (25th–75th percentile, colored box) and maxima/minima (whiskers). Source data are provided as a Source Data file.

Electrophysiological properties of cell types are often associated with morphological features. Motivated by differences in intrinsic membrane properties, we investigated morphological differences between EXC and FSI neurons in V1 and LPFC. For the EXC population, we reconstructed a total of 31 pyramidal cells (11 V1, 20 LPFC) and systematically compared their basal and apical dendritic trees (Fig. 6A, B). LPFC EXC cells exhibited significantly larger dendritic trees compared to V1. Total dendritic length was significantly greater in LPFC for both apical (2979 vs. 1159 µm, adj. *p* = 0.0010) and basal dendrites (2644 vs. 1489 µm, adj. *p* = 0.0023). Consistent with the larger size, LPFC pyramidal cells also showed higher branching complexity, measured by the number of dendritic endings, which were higher in apical (24.5 vs. 15, adj. *p* = 0.0010) and basal dendrites (PFC = 24.5, V1 = 18, adj. *p* = 0.0282) (Fig. 6B).

Given that pyramidal morphology is influenced by the radial position of the soma relative to the cortical surface, we next examined how soma depth correlates with dendritic features (Fig. 6C). We found no significant correlation between the number of endings (apical: τ = 0.192, adj. *p* = 0.2320; basal: τ = 0.143, adj. *p* = 0.3149), suggesting that branching complexity does not vary systematically across the cortical depth. In contrast, the total basal length showed a strong correlation (τ = 0.385, adj. *p* = 0.0179), indicating that basal dendrites grow longer with increased soma depth. Interestingly, this was not the case for total apical length (τ = 0.254, adj. *p* = 0.1342).

We also reconstructed a total of 14 FSI (4 V1 and 10 LPFC, one somatodendritic only) for comparison across cortical areas. FSI axons in LPFC were significantly larger than in V1, demonstrating divergence in both total length (19743 vs. 12239 µm, adj. *p* = 0.0497) and median path length (293.7 vs. 233.5 µm, adj. *p* = 0.0277). In contrast, dendrites total length (3695 vs. 3765 µm, adj. *p* = 0.5245) and median path length (LPFC: 175.5, V1: 126.8 µm, adj. *p* = 0.1385) were not significantly different across areas. The number of dendritic endings (# endings) was also not significantly different (22.5 vs. 32.5, adj. *p* = 0.0780).

## Discussion

We compared electrophysiological and morphological features of single neurons in two different areas of the common marmoset visual cortical processing stream, V1 and the LPFC^55–57^. We conducted intracellular recordings using whole-cell patch-clamp in acute brain slices and anatomical reconstructions of a subset of neurons in both areas. To identify e-types, we built a classifier trained on mouse data using anatomical and transgenic labels with e-features as predictors. We grouped the neurons into three major classes that we predicted the classifier could accurately identify using e-features: FSI, NFSI, and EXC. We then closely examined and compared e-features of the three e-types between areas V1 and LPFC. EXC cells in V1 were smaller, more excitable and fired narrower spikes than in LPFC. FSI in V1 also fired narrower spikes, but were less excitable than in the LPFC. NFSI shows spike widths and excitability in between EXC and FSI, but showed fewer differences between areas. Sag ratio was higher in V1 than in LPFC, for all e-types, but bursting was more prevalent in EXC, followed by NFSI and FSI neurons. In EXC and FSI neurons, bursting was more prevalent in LPFC than in V1, with a subset of LPFC FSI showing bursting. We also documented larger dendritic trees in EXC neurons and longer and more complex axons in FSI in LPFC relative to V1.

### Classifying neuron types using e-features

Neuronal diversity has been a puzzle for neuroscientists since the time of Santiago Ramon y Cajal, who described different cell morphologies across brain regions and species and suggested a relationship between the structure/morphology and function of neurons^58^. Indeed, morphology and intracellular electrophysiological measurements have been used to classify neurons^35^ and more recent progress in transcriptomics has considerably advanced this field^38,59–63^. Studies of e-features using large samples have mainly been conducted in mice, where transgenic tools for target-neuron labeling are available^51^. In primates, some studies have examined e-features and morphology of neurons mainly in the macaque monkey; they have highlighted common as well as distinctive features of cell types relative to rodents^15,17,18,27,43,44,64–68^. Such studies generally had a smaller sample size than rodent studies and/or were limited to a single cell type and/or brain area. However, alongside more recent studies of resected human tissue from patients with epilepsy or brain tumors, neuronal innovations found in primate studies highlight the importance of this topic in neuroscience research^69,70^, particularly for revealing vulnerabilities of primate brains to aging and diseases^71^.

Over the last decades, a small New World primate, the common marmoset, with shorter life and reproductive cycles than macaques, has opened the door to studies using larger samples, and has therefore acquired popularity in neuroscience research as a model species with relevance to the human^28–30,72,73^. Our study investigates e-type diversity in areas V1 and LPFC of the marmoset cortical visual pathways using the same protocols as the AIBS in mice and humans to minimize technical and methodological confounds when extrapolating measurements of e-features between datasets (e.g., marmoset and mouse data). Our classification using transgenic mouse labels, as well as anatomical features (e.g., spiny vs. aspiny) resulted in three main classes of neurons common to mice and primates^34,35,74^. We consider that EXC were mainly excitatory pyramidal cells, FSI were mainly PV + basket cells, and NFSI, comprised of VIP and SST (Calretinin + and Calbindin + in primates) neurons^34,35,37,42,46,64,74–77^.

Our tripartite classification may seem narrow in scope compared to the diversity of transcriptomic clusters identified in recent marmoset studies^60^. However, to the electrophysiologist, it is a tool that can be used to segregate these major functional groups when transgenic labels are unavailable. This is useful considering that EXC, FSI, and NFSI neurons have different functional roles and connectivity in circuits. EXC neurons in layer II/III are the main recipients of feedback inputs from and the origin of projections to other cortical areas. In V1, they diversify their responses relative to those of granular layer IV to become selective for combinations of single features (e.g., complex cells)^5^. In the LPFC EXC neurons in layers II/III form recurrent networks that maintain working memory signals in the absence of sensory inputs and send feedback signals to the rest of the brain for executive control^78–80^. FSI, mainly composed of basket and chandelier cells, receive inputs from EXC cells and target the same or other EXC cells’ bodies or proximal parts of their axons. They efficiently regulate response gain and synchrony within circuits^81,82^. NFSI comprise a very diverse group of cells that target dendritic compartments of EXC and other inhibitory neurons to regulate complex functions such as feature selectivity, center surround inhibition, and disinhibition^83–86^.

Further contributions of our study include highlighting homologies and differences between these broad categories of cell types in marmosets and providing a resource for further research and computational modeling, thereby advancing the use of these animals in research. The differences we observed across areas align with findings from transcriptomic studies^60^. One issue to overcome in future studies is that ours and other existing datasets are limited to a few brain areas (e.g., V1 in mice and temporal cortex in humans^51^, V1 and LPFC in marmosets (this study)), which makes it challenging to perform broad inter-area comparisons across species. Future work is needed to fill this gap and provide a comprehensive map of functional and anatomical properties of neurons across different cortical areas of primates.

### Differences in intrinsic properties and morphology between cell types in V1 and LPFC

A primary goal of this study was to identify distinctive features of cell types in areas V1 and the LPFC of the marmoset. V1 and LPFC are in ‘opposite’ extremes (beginning and end) of the primate cortical visual processing hierarchy^1^. V1 neurons must fast and reliably process incoming information from the thalamus. We found that EXC cells in V1 showed high excitability and narrow action potentials, which may contribute to the elevated firing rates and short spike latencies relative to downstream areas’ neurons reported by extracellular studies in macaques^87,88^. V1 also has the highest neuronal density along the visual cortical pathways; the small neuronal sizes may allow ‘packing’ more cells within the area receiving highly spatially-specific LGN inputs corresponding to small regions of the retina, resulting in a high-resolution map of the visual field^89^. We also found that V1 FSIs exhibit sustained high firing rates, low adaptation, high _Rin_(excitability), and low bursting. These features make V1 neurons suitable for linear or quasi-linear encoding of important spatiotemporal details of visual inputs.

On the other hand, LPFC EXC neurons have large dendritic arborizations, many spines^8,90^ and fire at lower rates than those in V1 and early sensory areas^91^. Importantly, firing rates in LPFC show a higher correlation with performance than in upstream areas such as MT^92^ and V1^93^. Thus, LPFC signals are more directly linked to our perceptions and actions than V1 signals. We found that EXC neurons in LPFC fired fewer spikes and had lower excitability (Rin) than those in V1, which may facilitate the selection of the stronger inputs corresponding to stimuli that are salient and/or behaviorally relevant^6^, producing sparse codes that underlie our perceptual capabilities and sense of awareness^94^. The same structural and functional complexity may make LPFC EXC cells vulnerable to new categories of disease that would affect the highly developed cognitive skills of primates, such as schizophrenia^95–97^ and Alzheimer’s disease^71,98^.

One finding of our study is the inversion of the relationship between EXC and FSI neurons’ excitability in the two areas. In V1, EXC neurons are more excitable than FSI neurons, while in LPFC, it is the reverse. FSI neurons receive their main inputs from local EXC cells. V1 may favor regimes of high excitation and less prominent inhibition, thereby registering ‘all’ visual inputs coming from the thalamus. Otherwise, the larger firing rates of EXC neurons could easily recruit FSI cells, so decreased excitability of the latter may be a mechanism to maintain excitation-inhibition balance within the local circuit. Conversely, LPFC FSIs are more excitable and therefore more sensitive to weaker inputs coming from low-firing-rate local EXC neurons. Additionally, LPFC EXC neurons with larger dendritic trees and FSI with longer axons allow communication over longer distances in the less densely populated region (relative to V1)^99^. EXC neurons and FSI interneurons intrinsic properties in V1 and LPFC may reflect the interactions between these cell types that are specific to each area.

Our results show that V1 neurons exhibit significantly larger HCN-mediated sag than lateral prefrontal cortex (LPFC) neurons across both excitatory and inhibitory cell types, indicating stronger Ih-current engagement in early visual cortex (a hyperpolarization-activated, mixed Na⁺/K⁺ inward current mediated by HCN channels that generates voltage sag, stabilizes membrane potential, limits temporal summation, and dynamically regulates neuronal excitability and timing in a state-dependent manner)^100^. In pyramidal neurons, HCN (Ih) channels sculpt dendritic synaptic integration and temporal summation, whereas in interneurons they regulate somatic excitability, spike timing, and network rhythmicity^54,101–103^. HCN channels are more abundant in human than in mouse cortical pyramidal neurons, indicating marked interspecies differences in I_h_ contribution^69^. We additionally found that HCN-mediated sag varies within primate visual processing areas across all neuronal types, indicating systematic intra-primate diversification of intrinsic excitability. Notably, the LPFC is thought to be particularly vulnerable to age-related reductions in HCN/I_h_, and the effects of aging further support the idea that HCN channels are dynamically regulated across the lifespan^71,104,105^. The mechanisms underlying this regulation, and how HCN expression and function vary with cell type, cortical area, and subcellular compartment (soma, dendrites, and axons) in primates, remain important open questions for future study.

The differences in sag could be linked to differences in input resistance. Input resistance reflects the total membrane conductance at rest, whereas sag reflects the activation of voltage-dependent inward currents during hyperpolarization, primarily the I_h_ current mediated by HCN channels. Neurons with stronger I_h_ typically show a larger depolarizing sag and a lower input resistance because I_h_ contributes to resting membrane conductance, leading to a frequent negative correlation between these measures across neurons or cortical areas^54^. However, they are not equivalent; input resistance can be low due to passive leak conductances without sag, and sag can be prominent even when input resistance is moderate. Sag can also influence other passive properties, such as the time constant^54^. Future studies should quantify the relationship between sag and other intrinsic properties in the marmoset by quantifying HCN channels in the different cell types.

Certain types of interneurons are more abundant in LPFC than in early sensory areas^46,77^. One issue that remains unclear is when and how such interarea differentiation of local circuitry and intrinsic neuronal properties occurs. For the case of LPFC, it is possible that the protracted development of primates provides a prolonged window to adjust mRNA and protein expression^106^ based on local feedback (i.e., levels of firing rate) and therefore produce variations of what have been considered canonical cell types of the neocortex. Species with less extended protracted developmental periods, such as mice, may have diversified less or differently, so neuronal types may appear more serially homologous^15,107^. This distinctive feature of primates may be highly relevant for the use of animal models to study human disease^108^ and therefore deserves further investigation.

### Bursting is a distinctive feature of LPFC neurons

A burst is a short, high-frequency train of spikes with a higher probability to elicit a postsynaptic spike via temporal summation of excitatory postsynaptic potentials than single spikes^109^. Bursts could arise by intrinsic mechanisms or by network dynamics^109^. Intrinsic bursters usually express certain conductances, such as T-type calcium channels and show a slow depolarization mechanism on top of which action potentials are generated^110,111^. Bursting in EXC cells has been reported in macaque LPFC^27^, suggesting that it is a feature of this area in primates. Importantly, in some LPFC FSI neurons, current injections elicited depolarizing events with slow kinetics that led to long latencies of action potentials evoked at low rheobase, suggesting that certain FSI in LPFC showed comparable bursting behavior to EXC cells.

Bursting has been associated with a variety of functions, such as an increase in the efficiency of synaptic transmission^112,113^ and modulation of synaptic plasticity by increasing temporal summation of EPSPs and long-term potentiation (LTP) in postsynaptic terminals^114–116^. The increase in bursting in LPFC relative to V1 may be linked to the ability of LPFC neurons to become tuned to novel objects flexibly^6,117,118^. It is possible that neurons in early areas such as V1 consistently process basic visual features (high stability - low plasticity), which is necessary for encoding the same sensory information (e.g., color, orientation) stably, regardless of whether it belongs to familiar or novel objects. On the other hand, neurons in the LPFC rapidly ‘learn’ new feature associations (low stability - high plasticity), which is necessary when encountering novel complex objects. Previous studies have reported a gradient in the ratio of NMDA to AMPA receptors^119,120^ along the hierarchy of visual processing, with LPFC showing a higher ratio compared to V1. Combined with intrinsic bursting, NMDA gradients can enhance LPFC ability to build selectivity for new categories or rules^121–123^.

Our results are compatible with a recently proposed model of intrinsic time scales in the hierarchy of sensory-motor processing in the marmoset. Early sensory areas such as V1 have short timescales that enable them to respond rapidly and effectively to incoming stimuli, whereas association areas such as the LPFC display longer timescales that support the integration of information over a relatively long-time window, more compatible with the scales of perception and behavior^124^. Our results can also be interpreted within the framework of the feature integration theory of perception and attention^125–127^, in which essential features are pre-attentively processed in early visual areas such as V1 and are later assembled for conscious processing by mechanisms of attention in the LPFC^6^.

### Serial homology and neuronal diversity in the primate visual system

It has been proposed that, in the mammalian neocortex, cell types follow the principle of *serial homology*: cell types across brain areas are variations on a common theme organized in a similar basic pattern. Differences between serially homologous structures are typically quantitative rather than qualitative^107^. However, much of the data supporting this hypothesis has been collected in a single species, the mouse, and in sensory areas (e.g., barrel cortex or V1)^128^. Mice do not show the stark differences in EXC cell morphology between V1 and PFC (Gilman et al., 2016); thus, there may appear more serial homologs than in primates. In primates, previous studies have observed differences in structural complexity, ranging from branching patterns to increases in the number of dendritic spines^15,17,20,21,23,25–27,71,76,119^ between V1 and LPFC neurons. Moreover, we observed qualitative differences in e-features across cell types, including the emergence of intrinsic bursting in LPFC FSIs. Thus, primate brains with expanded neocortices have diversified single-neuron structure and function, achieving greater computational power and behavioral complexity, which may have played a role in the emergence of primate complex cognition.

Cortical inhibitory interneurons have been classically considered less diverse across areas and species^34^. However, most of what we know about interneuron diversity is from studies in mice. Interestingly, a recent report has shown substantial variation in the expression of different types of mRNAs across various species (mice, ferrets, marmosets and humans^60^. In agreement with our results, several transcripts were differentially expressed in primate interneurons depending on the area^60^, supporting the notion of existing qualitative differences in interneuron subtypes across areas in primates. There has been a notion that major features of interneuron types are preserved across mammals^34,35,129^. This seems certain to a degree. However, our results indicate nuanced inter-area differences in specific interneuron features that might be revealed by sampling large populations. We propose that neuronal diversity across brain areas may be a hallmark of primate evolution and may arise during protracted periods of brain development^130^. How this diversification of cell types has impacted normal brain function and vulnerability to new categories of brain diseases affecting humans^131^ is an open question.

Our study revealed consistent differences in the e-features and morphology of excitatory and inhibitory neurons between areas V1 and LPFC of a New World primate, the common marmoset. Thus, area-dependent cell type specialization is likely to be a feature of the primate visual system that extends to inhibitory cell types and across species. Our study also provides a resource for exploring and modeling cell types’ electrophysiological and morphological features in primates (www.primatedatabase.com), as well as for comparative studies across species.

## Methods

### Statement on animal research

Research on NHPs is ethically sensitive but currently irreplaceable in neuroscience research. The authors of this study are committed to pursuing the best scientific result with the least possible harm to the animals. Data were collected from 51 (male: 33, female: 18) common marmosets (26 at Western University + 25 at University of Göttingen) with a median age of 5.08 years (IQR: 4.81, min: 1.52, max 18.06). Animal research from the Canadian group was conducted in accordance with the Canadian Council of Animal Care policy on the care and use of laboratory animals. The experiments were approved by the Animal Care Committee of the University of Western Ontario. Animals from the German research group were fostered and kept at the German Primate Center, Göttingen, Germany. Husbandry and experiments were conducted in compliance with Directive 2021/63/EU of the European Union and the German Animal Welfare Act and therefore meet the regulations of the European Animal Research Association. The animals were sacrificed as part of a broad study at the German Primate Center approved by the Lower Saxony State Office for Consumer Protection and Food Safety (LAVES; reference number 33.19-42502-04-20/3458). Experimental procedures were in accordance with the regulations of the German Animal Welfare Act. All animals were under constant veterinary supervision.

### Acute marmoset ex vivo brain slice preparation

Marmosets were anesthetized with Ketamine (20 mg/kg, intramuscular) and isoflurane (2–5%) and then euthanized by an overdose of pentobarbital. In the latter case, an ice-cold solution was poured over the head before opening the skull to slow down cellular processes and decrease brain tissue deterioration during organ removal. Afterwards, the brain was immediately rinsed with pre-chilled (2–4 °C) N-methyl-D-glucamine (NMDG) substituted artificial cerebrospinal fluid (aCSF) containing 92 mM NMDG, 2.5 mM KCl, 1.2 mM NaH_2_PO_4_·H_2_O, 30 mM NaHCO_3_, 20 mM 4-(2-hydroxyethyl)−1-piperazineethanesulfonic acid (HEPES), 25 mM glucose, 5 mM Na-ascorbate, 2 mM thiourea, 3 mM Na-pyruvate, 10 mM MgSO_4_·7H_2_O and 0.5 mM CaCl_2_·2H_2_O. The pH of the aCSF was adjusted to 7.3–7.4 with concentrated hydrochloric acid (37%), and the osmolality was between 300–305 mOsm/kg. The solution was oxygenized with 95% O_2_ and 5% CO_2_ (carbogen) for 15 min prior to animal surgery. The brain was transferred into a container containing the same NMDG aCSF and quickly transported from the animal facility to the laboratory room (10–20 min)^51,132^.

The hemispheres were separated and trimmed to blocks of prefrontal and visual cortex. Slices of 300 µm thickness were cut with a vibratome VT1200 S (Leica) using the same ice-cold NMDG aCSF as above. Subsequently, the slices were transferred to a recovery chamber filled with NMDG aCSF at 32 °C and incubated for 12 minutes before being stored in a holding chamber at room temperature. This chamber was filled with HEPES aCSF containing 92 mM NaCl, 2.5 mM KCl, 1.2 mM NaH_2_PO_4_·H_2_O, 30 mM NaHCO_3_, 20 mM HEPES, 25 mM glucose, 5 mM Na-ascorbate, 2 mM thiourea, 3 mM Na-pyruvate, 2 mM MgSO_4_·7H_2_O and 2 mM CaCl_2_·2H_2_O. The slices were stored for up to 10 h with minimal submersion and transferred to fresh HEPES aCSF after 6 h. HEPES provided additional support to pH buffering and reduced slice deterioration over extended time periods. All solutions were continuously oxygenated with carbogen gas and set to 300–310 mOsm/kg.

The solutions for ex vivo brain slice preparation and electrophysiological recordings were adopted from the technical white paper by the Allen Institute for Brain Science (AIBS) Cell Type Database. The latter facilitates the comparison between our marmoset dataset with mouse and human datasets from AIBS Cell Type Database^51,69,70^.

### Patch clamp electrophysiology

Individual brain slices were transferred into the recording chamber and continuously perfused with ~ 32 °C carbogen-saturated aCSF containing 126 mM NaCl, 2.5 mM KCl, 1.25 mM NaH_2_PO_4_·H_2_O, 26 mM NaHCO_3_, 20 mM HEPES, 12.5 mM glucose, 1 mM MgSO_4_·7H_2_O, 2 mM CaCl_2_·2H_2_O and a mix of synaptic blockers (2 mM kynurenic acid and picrotoxin 0.1 mM). Thick-walled borosilicate glass pipettes were manufactured on the day of recording and filled with intracellular solution containing 126 mM K-gluconate, 4 mM KCl, 0.3 mM EGTA, 10 mM HEPES, 4 mM K_2_-ATP, 0.3 mM Na-GTP, 10 mM Na_2_-Phosphocreatinine. The pH was adjusted to 7.3–7.4 with concentrated KOH, and the osmolality was set to 290 mOsm/kg with sucrose when necessary. The internal solution was supplemented with 0.5% biocytin to fill the recorded neurons for histology. Pipette resistance in the bath was 3-7 MΩ. Recording equipment varied across experimenters: data were acquired with either a Multiclamp 700B and a Digidata 1440 (Axon instruments/Molecular Devices), a SEC-10LX or SEC-05X (NPI electronics) digitized with a 1401-3 A (CED) or an EPC 10 USB (HEKA Elektronik). The data was recorded with pCLAMP 10&11 (Molecular Devices), Signal 5&7 (CED) and Patchmaster 2x92 (HEKA Elektronik). After forming a stable seal above 1 GΩ and break-in, access resistance was determined in voltage-clamp (VC) mode. Then, the recording mode was switched to current clamp (CC) and bridge balance and pipette capacitance compensation procedures were applied. When necessary, a holding current was applied and continuously readjusted throughout to maintain a membrane potential close to the initial value after break-in. Cells that required more than 20 pA holding current to be kept at − 60 mV in the initial whole-cell configuration were not recorded from. Recording protocols and procedures were standardized across laboratories and acquisition equipment. Neurons were subjected to hyper- and depolarizing 1 s square pulse current injections.

### Histology

After recording, slices were fixed with 4% m/v PFA + 15% v/v picric acid at 4 °C for a period of 24–36 h and then washed in phosphate-buffered saline (0.01 M, pH 7.4; PBS). Then, slices were permeabilized by incubation in PBS + 2% Triton-X for 2 × 15 min. Biocytin filling of patched cells was made visible by a 4 h incubation of streptavidin-Alexa633 (1:300, S21375k Invitrogen by Thermo Fisher Scientific). After a subsequent washing step, slices were incubated in PBS with DAPI (1:4000, D1306, Invitrogen by Thermo Fisher Scientific). Finally, slices were mounted on specimen slides with aqua-polymount (Polysciences, PA, USA).

Imaging was done using a SP8 confocal microscope (Leica, LAS X) or an LSM 880 with Airyscan (Zeiss, ZenBlack 2.3) to assess the cell location and quality of the filling. Cells with sufficiently retained dendritic and axonal arborization were used for another acquisition with 40 x or 63 x magnification (z-step size 0.5–0.8 µm). These image stacks were then used to reconstruct the neuronal morphology using Neurolucida 360 2021.1.3 (MBF Bioscience). Automated quantifications of neurites were obtained with Neurolucida explorer (MBF Bioscience).

There are three different types of spiny neurons in the cortex: pyramidal cells, spiny-stellate cells and star-pyramidal cells, which differ in their soma shape and dendritic configuration^133^. However, these features become less clear to differentiate in the upper layer 2 due to the condensed nature of this cell layer. For example, the length and orientation of primary dendrites do not always follow the characteristic patterns of these cell types in deeper layers. Thus, for this study, we pooled the three cell types and analyzed their morphology collectively as spiny pyramidal cells (tagged as excitatory, EXC). For non-spiny or sparsely spiny non-pyramidal neurons (tagged as inhibitory cells), we used established criteria to assign them to a morphological cell type^35,42,66^.

### Analysis of electrophysiological features

The recordings were converted into the Neurodata Without Borders (nwb) format (version 2.4.0^134^) using the MATNWB repository. Additional information, such as subject data (age, sex, etc.), acquisition parameters (access resistance, temperature, etc.) or histological specifications (layer, morphology, etc.), was added if available. A custom MATLAB analysis pipeline was used for quality control and extraction of electrophysiological features. The code is publicly available on GitHub (GitHub - mfeyerab/MATFX). The quality control routine is described in short here: cells were not considered for further analysis if one of the following criteria was met: 1) an initial membrane potential above − 55 mV, or 2) input resistance or action potential (AP) waveform features could not be determined due to lack of data or excessive noise. In addition, recording quality was assessed in a sweep-wise fashion. For this purpose, a time window of at least 250 ms for each pre- and post-stimulus period was selected. A sweep failed quality control if one of the following criteria were met: (1) the membrane potential in either segment displayed a root mean square error of 0.65 mV or more, (2) the membrane potential difference between pre- and post-stimulus periods exceeds 2.5 mV, (3) the membrane potential in the pre-stimulus segment is more depolarized than − 52.5 mV, (4) the membrane potential of the pre-stimulus deviates more than 6 mV from the median pre-stimulus membrane potential of all passing sweeps, (5) sweeps were flagged by manual curation based on evaluation of the pre-stimulus test pulse (see Table 1 and Supplementary Table 1**for a list of key features and values**, and Supplementary Table 2 for a description of all electrophysiological features). For each cell, we measured burst as 1 minus the ratio of the first ISI divided by the mean of the remaining ISIs from the hero sweep (Burst Index). Strongly bursting cells will show a burst index close to 1, while weakly bursting cells will show a burst index closer to 0 (see example cell in Fig. 5C).

### UMAP and cross-species classification

Uniform Manifold Approximation and Projection, UMAP^135^ space was created by combining three datasets, each obtained from a different species. Mouse and human data were available at the AIBS Cell Type Database. For the classification analyses, separate training and test datasets were used for each species. Cells with a missing value in more than 25% of features were excluded from the subsequent analysis, yielding a total number of 2766 cells (1909 mouse, 401 human, and 464 marmoset). Initially, we selected 29 electrophysiological features (see supplement for more details). These features were reduced to 16 components by probabilistic PCA, explaining more than 95% of the total variance. Then data was transformed into UMAP space using custom MATLAB scripts in two instances: (i) in a space with two components for visualization of electrophysiological diversity in the different datasets (number of nearest neighbors = 199, minimum distance = 0.2) and (ii) in a space with three components for supervised classification across species (number of nearest neighbors = 199, minimum distance = 0.25).

For classification, three electrophysiological labels (e-types) were chosen: Fast-spiking interneurons (FSI), non-fast-spiking interneurons (NFSI), and excitatory cells (EXC). Ground truth data (Supplementary Fig. 1) were obtained from Cre reporter lines and electrophysiological types (e-types, determined by unsupervised clustering by Gouwens et al. 2019). Cells from the mouse cortex that expressed Cre under the parvalbumin (PV) promoter and an e-type ranging from Inh_8 to Inh_13 were considered FSI. Cells that expressed Cre under a somatostatin (SST) promoter and an e-type ranging from Inh_4 to Inh_7 were considered SST cells, whereas cells that expressed Cre under a serotonin receptor 3a (5-HT3A) or vasoactive (poly)peptide (VIP) promoter and had either e-type 2 or 3 were considered VIP cells. Lastly, cells that had the e-type “Inh_1”, which was marked by low spike-frequency adaptation and commonly high latency of the rheobase spikes, and expressed Cre under either a 5-HT3A or a neuron-derived neurotrophic factor (NDNF) promoter, were considered neurogliaform (NGF) cells. NFSI consisted of the SST, VIP and NGF subtype, which do not show the distinctive fast-spiking (FS) phenotype of PV cells. Cells that had a spiny dendritic type were considered EXC. Cell type composition of training data was informed by histological data of the primate neocortex (see marmoset gene atlas^136^;), consequently the ratio of SST, VIP and NGF cells within the training data was fixed to 2:2:1. A random forest classifier for cross species cell labeling was trained^137^ with uniform priors within a subset of the 3 component UMAP data consisting of 1191 mouse cells with fixed cell type proportions (see Fig. 3A) drawn from a pool of 1462 cells with ground truth labels. Similarly, a test data set of 150 cells was drawn from the same pool. To evaluate the performance of the classification model on non-mouse cells, we use a small subsample of morphologically identified cells for the marmoset and human, which could be assigned a morphological truth label similar to the mouse data. Consequently, additional “test” samples were generated for human and marmoset data with cell class composition representative of the entire data set (see Fig. 3A and Supplementary Fig. 4A). This process was repeated 500 times with different training and test data selections. Test data were excluded from the initial UMAP embedding and transformed separately.

### Statistical analysis

All hypothesis testing regarding differences between areas was done with two-sided rank sum tests in MATLAB, and effect sizes were reported by rank bi-serial correlation (r_rb_). All r_rb_ values and their 95% confidence intervals were calculated with the measures-of-effect-size-toolbox^138^. Reported *p*-values were corrected for false discovery rates via the Benjamini-Hochberg procedure. Correlations reported were either tested by Spearman (correlation coefficient: ρ), Kendall’s tau in case of *n* < 30 (correlation coefficient: τ) or Pearson (correlation coefficient: r) procedures.

The residual values of rheobase were obtained from a linear regression model with rheobase as the dependent variable and the ratio between the difference in the action potential threshold and the resting membrane potential (ΔV), and the input resistance (R) as predictor variables. This model (Rheo = β0 + β1*(ΔV/R_inHD_) + residuals (ε)) captures the passive/ohmic aspect of the rheobase.

### Effect of different acquisition systems on AP waveform

Our data were recorded at two different sites with different acquisition systems. Therefore, we examined whether this could have influenced electrophysiological measurements. Data recorded with the HEKA amplifier show significantly higher AP amplitudes in aspiny cells than those recorded with the npi amplifier (Supplementary Fig. 2D, E). We believe that this difference arises from the varying effectiveness of compensating the pipette capacitance. Uncompensated pipette capacitance, together with the access resistance, create a passive low-pass filter which artificially shorten and widen the AP. This effect was stronger in neurons with narrow spike waveforms. The selection of AP waveform features for the UMAP procedure was informed by these observations. AP width was the only wave form parameter chosen as input, since it showed no significant effect between the amplifier systems (Supplementary Fig. 2E). Ultimately, the UMAP projection did not suggest clustering of neurons by acquisition equipment (Supplementary Fig. 2A), and we conclude that our intrinsic property profiles are mainly shaped by biological factors such as cell identity.

### Reporting summary

Further information on research design is available in the Nature Portfolio Reporting Summary linked to this article.

## Supplementary information

Supplementary Information

Reporting Summary

Transparent Peer Review file

## Source data

Source Data

## Data Availability

The marmoset data generated in this study have been deposited in the online database under primatedatabase.com Link. The processed marmoset data generated in this study are provided in the Supplementary Information/Source Data file. The AIBS mouse/human data used in this study are available in the AIBS database under celltypes.brain-map.org Link Source data are provided in this paper.

## Code availability

The MATLAB code for quality control and extraction of electrophysiological features is publicly available on GitHub (https://GitHub-mfeyerab/MATFX).
