# Supplementary material for: Intrinsic neuronal diversity as a substrate for cortical area specialization in primate vision
Source: Nat Commun. 2026 Jun 9;17:7335. doi: 10.1038/s41467-026-73734-5 (PMC13402634; doi:10.1038/s41467-026-73734-5)
Supplement: Supplementary file 1 — Supplementary Information [file 41467_2026_73734_MOESM1_ESM.pdf]

Intrinsic Neuronal Diversity as a Substrate for Cortical Area Specialization in Primate Vision – Supplementary Information

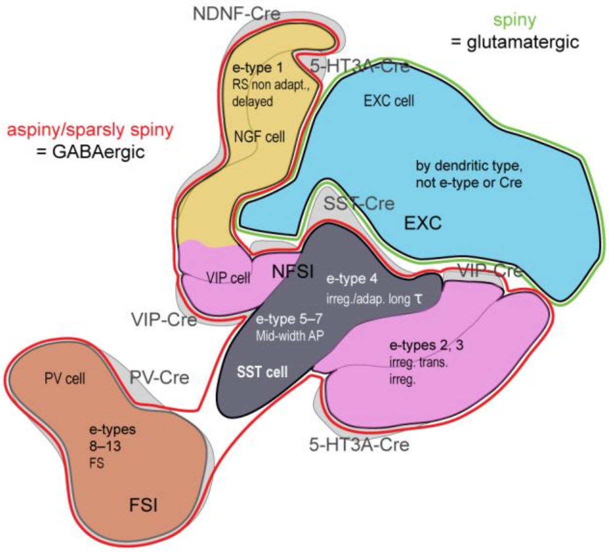

Supplementary Figure 1. Class selection criteria for the UMAP classifier
Criteria for training data for FSI, NFSI and EXC cells based on Cre-dependent mouse lines and e-types from the AIBS dataset. Source data are provided as a Source Data file.

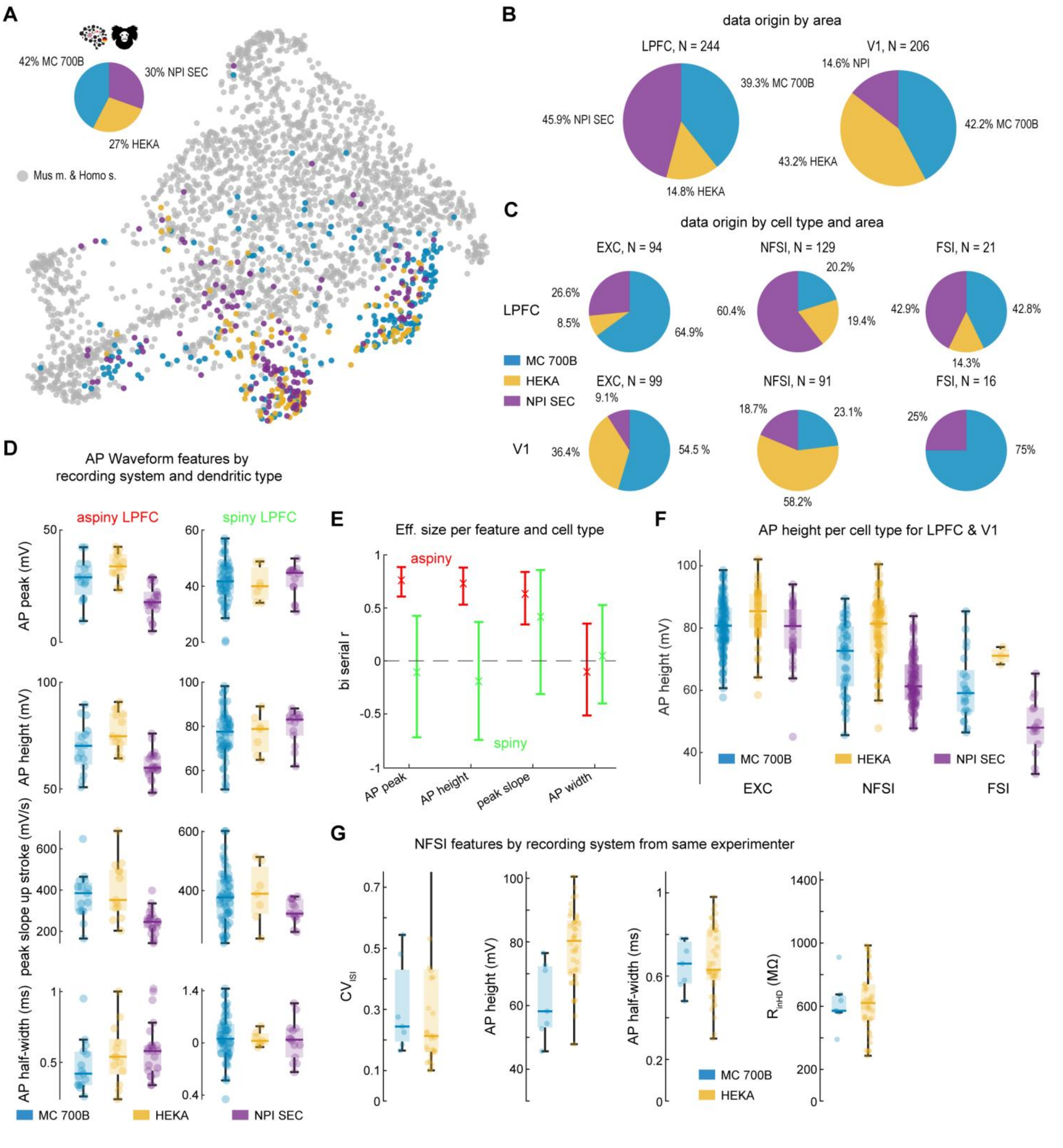

Supplementary Figure 2: Effects of recording system on electrophysiological features
A: Common UMAP with all recorded data: blue, Multiclamp (MC) 700B, yellow, HEKA, purple, npi SEC. The pie-chart shows proportions recorded in each system. **B:** Data origin split by recording system and recorded area. **C:** Data origin split by recording system, recorded area and cell type. **D:** Comparison of AP waveform features by dendritic type (left: aspiny LPFC, right: spiny LPFC): MC 700B (spiny: n = 56, aspiny: 15), HEKA (spiny: n = 7, aspiny: n = 13), npi SEC (spiny: n = 14, aspiny: n = 22). **E:** Comparison of effect sizes across features with 95% confidence interval between HEKA and npi per feature and dendritic type (red: aspiny, green: spiny) based on the data shown in D. The first three features were excluded as input for UMAP projection due to a noticeable effect in aspiny cells caused by amplifier type**.** Only AP half-width was included. **F:** Comparison of AP height for each recording system and cell type from both areas combined. MC 700B: EXC n = 115, NFSI n = 47, FSI n =

21; HEKA: EXC n = 44, NFSI n =78, FSI n = 3; NPI SEC: EXC n = 34, NFSI n = 94, FSI n = 14) **G:** Comparison of electrophysiological features of NFSI for different recording systems from the same experimenter. MC 700B n = 7; HEKA n = 42 All boxplots show median (colored line), IQR (25th–75th percentile, colored box) and maxima/minima (whiskers). Colored circles are outliers. Source data are provided as a Source Data file.

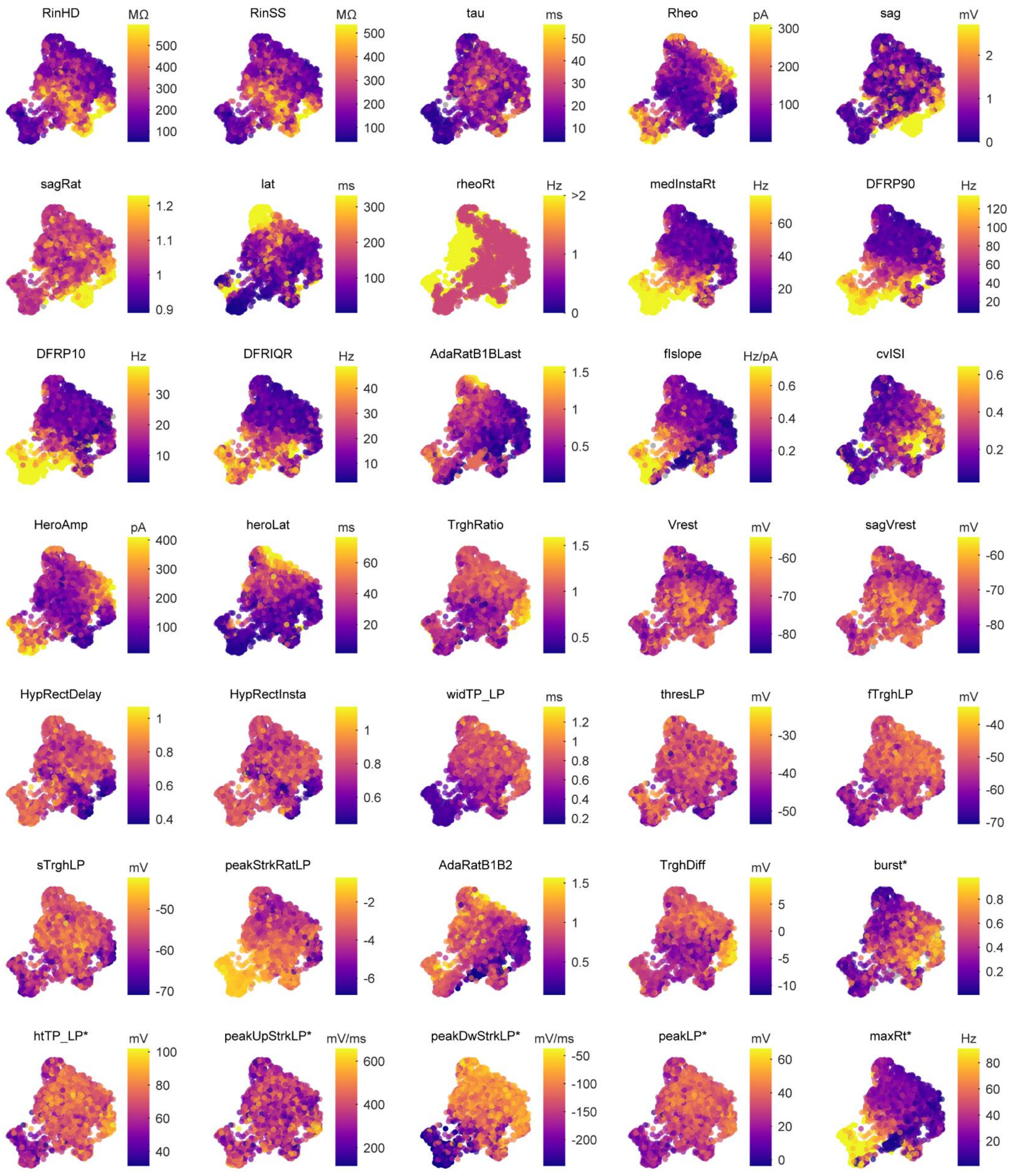

Supplementary Figure 3: UMAPs of mouse and marmoset e-features color-coded RinHD: input resistance based on highest deflection. RinSS: input resistance based on steady state. tau: membrane time constant. Rheo: rheobase. sag: sag difference in membrane potential. sagRat: sag ratio. lat: latency of first spike. rheoRt: firing rate of rheobase sweep. medInstaRt: median instantaneous firing rate. DFRP90: dynamic frequency range, 90th percentile. DFRP10 dynamic frequency range, 10th percentile. DFRIQR: dynamic frequency range, interquartile range. AdaRatB1BLast: adaptation ratio, first bin versus last (thirteenth) bin. fIslope: frequency-current curve slope. cvISI: coefficient of variance of ISI. HeroAmp: current

amplitude of hero sweep. heroLat: latency of first spike at hero sweep. TrghRatio: trough ratio. Vrest: resting membrane potential. sagVrest: sag resting membrane potential. HypRectDelay: delayed rectification at most hyperpolarizing sweep. HypRectInsta: instantaneous rectification at most hyperpolarizing sweep. widTP_LP: action potential half-width. thresLP: threshold of action potential. fTrghLP: fast trough. sTrghLP: slow trough. peakStrkRatLP: peak stroke ratio. AdaRatB1B2: adaptation ratio, first bin versus second bin. TrghDiff: trough difference. burst: burst index. htTP_LP: action potential height. peakUpStrkLP: peak upstroke. peakDwStrkLP: peak downstroke. peakLP: peak of action potential. Gray represents cells with missing features. Features with asterisk where not included in UMAP generation. Source data are provided as a Source Data file.

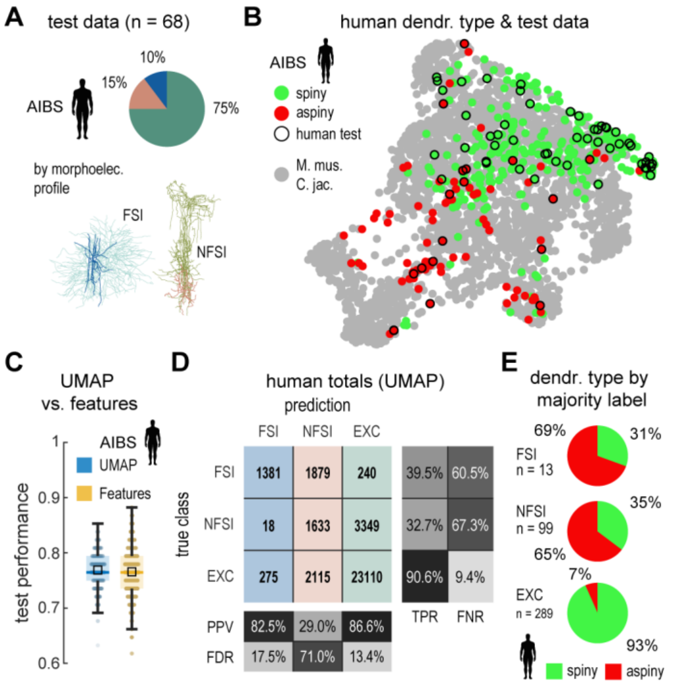

Supplementary Figure 4: Classification of human cell types

A: Pie chart showing cell type composition of human test data. Three different classes were used: FSI (blue), NFSI (ocher) and EXC (green), with example morphologies of FSI cells (left side, dark blue dendrites) and NFSI (right side, ocher dendrites). **B:** Example UMAP visualization with dendritic type of human cells (spiny, green; aspiny, red) and example test dataset (black circle) with collective mouse and marmoset cells. **C:** Performance of classifier across repetitions based on the UMAP (blue) and electrophysiological features (yellow). Performance of classifier for UMAP- or e-feature-approach (29 features) across repetitions. Boxplots show median (colored line), IQR (25th–75th percentile, colored box) and maxima/minima (whiskers). Black rectangles show the mean performance. Median test performance was 76.5% vs. 76.5% (UMAP vs. features). D: Confusion matrices for human test data showing all classification totals. PPV = positive predictive value, FDR = False Discovery Rate, TPR = True Positive Rate, FNR = False Negative Rate. E: Pie charts showing proportion of dendritic type per majority classifier prediction. Source data are provided as a Source Data file.

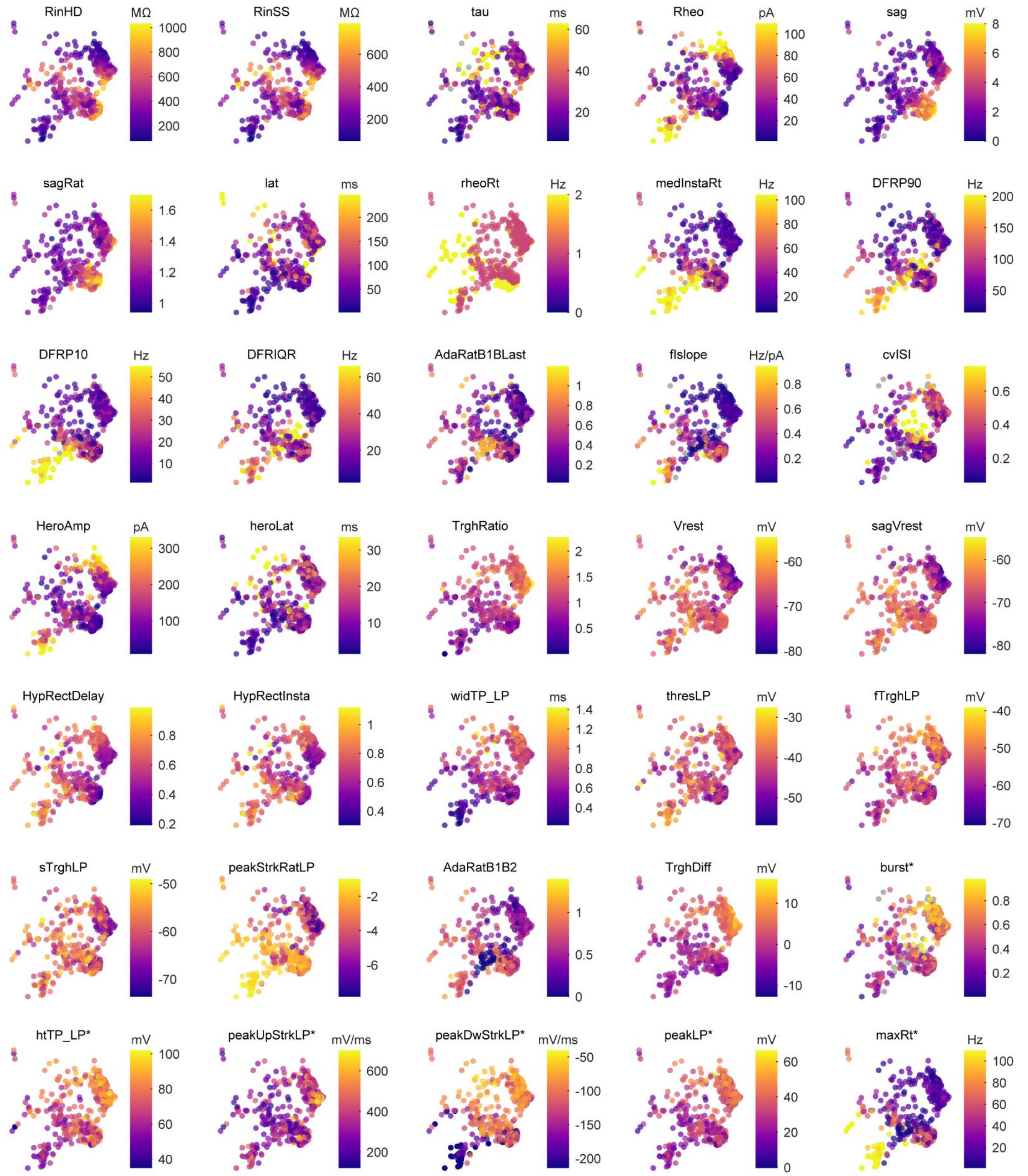

Supplementary Figure 5: UMAPs of marmoset e-features color-coded
RinHD: input resistance based on highest deflection. RinSS: input resistance based on steady state. tau: membrane time constant. Rheo: rheobase. sag: sag difference in membrane potential. sagRat: sag ratio. lat: latency of first spike. rheoRt: firing rate of rheobase sweep. medInstaRt: median instantaneous firing rate. DFRP90: dynamic frequency range, 90th percentile. DFRP10 dynamic frequency range, 10th percentile. DFRIQR: dynamic frequency range, interquartile range. AdaRatB1BLast: adaptation ratio, first bin versus last (thirteenth) bin. fIslope: frequency-current curve slope. cvISI: coefficient of variance of ISI. HeroAmp: current

amplitude of hero sweep. heroLat: latency of first spike at hero sweep. TrghRatio: trough ratio. Vrest: resting membrane potential. sagVrest: sag resting membrane potential. HypRectDelay: delayed rectification at most hyperpolarizing sweep. HypRectInsta: instantaneous rectification at most hyperpolarizing sweep. widTP_LP: action potential half-width. thresLP: threshold of action potential. fTrghLP: fast trough. sTrghLP: slow trough. peakStrkRatLP: peak stroke ratio. AdaRatB1B2: adaptation ratio, first bin versus second bin. TrghDiff: trough difference. burst: burst index. htTP_LP: action potential height. peakUpStrkLP: peak upstroke. peakDwStrkLP: peak downstroke. peakLP: peak of action potential. Gray represents cells with missing features. Features with asterisk where not included in UMAP generation. Source data are provided as a Source Data file.

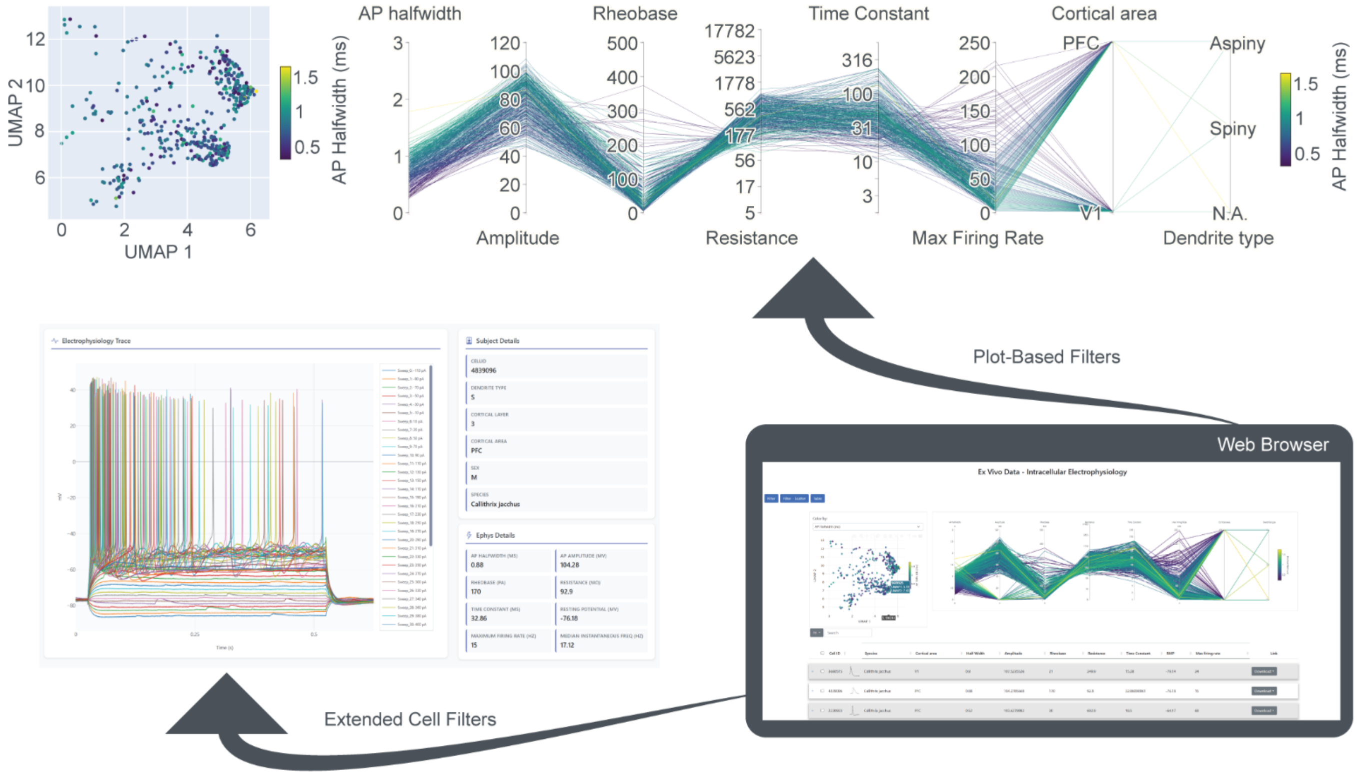

Supplementary Figure 6: Example of the primate cell type database resource. https://primatedatabase.com/

Supplementary Table1: Electrophysiological features for cell type cross area comparison

Feature	Cell	p_{raw}-value	z-value	ranksum	LPFC n	V1 n	effect size	eff. size CI1	eff. size CI2	V1 median	V1Q1	V1Q3	LPFC median	LPFC Q1	LPFC Q3	p_{corr}-value
R_{in} (MΩ)	EXC	5.3e-21	-9.404	5470	94	99	0.679	0.593	0.753	463.500	336.075	588.250	205.250	167.000	287.500	**1.6e-20**
R_{in} (MΩ)	NFSI	0.001	-3.252	12576.5	128	91	0.220	0.097	0.344	576.000	437.750	716.175	467.300	328.450	665.350	**0.002**
R_{in} (MΩ)	FSI	0.037	2.084	500	22	16	-0.345	-0.621	-0.049	184.300	139.400	234.950	243.500	191.900	326.900	**0.037**
sag Ratio	EXC	3.2e-6	-4.658	6927.5	91	99	0.339	0.207	0.468	1.220	1.150	1.308	1.130	1.090	1.188	**9.6e-6**
sag Ratio	NFSI	0.006	-2.763	12472.5	126	91	0.188	0.052	0.318	1.400	1.195	1.658	1.295	1.160	1.450	**0.009**
sag Ratio	FSI	0.030	-2.172	347.5	22	15	0.365	0.070	0.635	1.130	1.105	1.165	1.100	1.080	1.120	**0.030**
Rheo (pA)	EXC	3.7e-21	9.439	12768.5	94	99	-0.681	-0.754	-0.599	25.000	10.000	35.750	85.000	50.000	110.000	**1.1e-20**
Rheo (pA)	NFSI	0.414	0.817	14455.5	128	91	-0.055	-0.184	0.076	25.000	10.000	40.000	30.000	10.000	50.000	0.414
Rheo (pA)	FSI	0.021	-2.315	350.5	22	16	0.383	0.066	0.681	175.000	95.000	247.500	77.500	70.000	110.000	**0.031**
AP_{width} (ms)	EXC	4.9e-6	4.567	10888.5	94	99	-0.330	-0.455	-0.195	0.800	0.705	0.900	0.920	0.800	1.040	**1.5e-5**
AP_{width} (ms)	NFSI	0.105	-1.621	13331	128	91	0.110	-0.026	0.246	0.640	0.540	0.820	0.600	0.520	0.710	0.105
AP_{width} (ms)	FSI	0.001	3.361	542	22	16	-0.555	-0.756	-0.315	0.270	0.240	0.280	0.340	0.300	0.400	**0.001**
tau (ms)	EXC	0.225	-1.213	8421.5	93	97	0.088	-0.056	0.225	27.940	23.455	38.803	27.040	20.760	35.800	0.338
tau (ms)	NFSI	0.132	-1.504	11730	120	85	0.105	-0.029	0.232	24.240	17.155	36.460	21.570	14.560	33.390	0.338
tau (ms)	FSI	0.710	0.371	430.5	22	15	-0.064	-0.398	0.271	12.600	9.335	17.175	13.520	11.320	16.140	0.710
burst index	EXC	0.012	2.516	6119	70	84	-0.204	-0.357	-0.048	0.643	0.489	0.761	0.740	0.540	0.873	**0.018**
burst index	NFSI	0.809	-0.242	11158	119	69	0.018	-0.128	0.164	0.457	0.341	0.601	0.482	0.369	0.583	0.809
burst index	FSI	0.002	3.030	532	22	16	-0.501	-0.730	-0.224	0.330	0.201	0.354	0.433	0.345	0.546	**0.007**
adap. index	EXC	0.003	2.963	10267	94	99	-0.214	-0.346	-0.077	0.670	0.500	0.860	0.775	0.650	0.920	**0.009**
adap. index	NFSI	0.142	-1.468	13402	128	91	0.099	-0.037	0.230	0.510	0.373	0.790	0.500	0.355	0.670	0.142
adap. index	FSI	0.008	-2.663	338.5	22	16	0.440	0.151	0.673	0.585	0.485	0.890	0.460	0.280	0.580	**0.012**
RMP (mV)	EXC	0.780	0.280	9227	94	99	-0.020	-0.161	0.120	-72.130	-75.388	-68.545	-72.595	-74.940	-68.340	0.780
RMP (mV)	NFSI	0.007	2.715	15335	128	91	-0.184	-0.322	-0.047	-66.030	-68.145	-64.185	-65.215	-65.990	-64.180	**0.020**
RMP (mV)	FSI	0.416	-0.813	401	22	16	0.136	-0.185	0.448	-64.770	-67.005	-61.785	-65.775	-69.930	-63.070	0.624
Latency (ms)	EXC	0.902	0.124	9166.5	94	99	-0.009	-0.153	0.137	90.780	60.385	171.445	102.220	73.700	125.600	0.902
latency (ms)	NFSI	0.756	0.311	14224	128	91	-0.021	-0.158	0.114	62.980	30.030	131.815	60.340	39.440	97.860	0.902
latency (ms)	FSI	0.001	3.341	542.5	22	16	-0.552	-0.774	-0.287	14.300	11.850	19.790	34.160	22.440	42.460	**0.003**
medInstaRt (Hz)	EXC	0.004	-2.879	6529.5	84	94	0.216	0.067	0.359	22.345	19.090	26.610	19.545	15.705	24.840	**0.012**
medInstaRt (Hz)	NFSI	0.075	-1.781	12421.5	124	88	0.123	-0.014	0.256	53.040	38.025	68.690	46.065	31.430	64.535	0.075
medInstaRt (Hz)	FSI	0.026	-2.232	353	22	16	0.369	0.051	0.652	127.555	112.845	153.845	112.995	99.300	122.850	**0.038**
DFRP90 (Hz)	EXC	0.582	-0.551	7328.5	84	94	0.042	-0.101	0.191	37.870	31.200	55.090	38.220	28.505	50.125	0.582
DFRP90 (Hz)	NFSI	0.001	-3.395	11711.5	124	88	0.234	0.102	0.358	87.280	69.725	140.400	74.245	50.765	99.570	**0.002**
DFRP90 (Hz)	FSI	0.046	-1.996	361	22	16	0.331	0.005	0.618	178.775	149.020	197.380	152.010	132.630	169.490	0.069
DFRIQR (Hz)	EXC	0.431	-0.787	7247.5	84	94	0.059	-0.090	0.208	8.800	6.950	13.370	8.410	6.805	12.970	0.451
DFRIQR (Hz)	NFSI	3.1e-5	-4.170	11370.5	124	88	0.287	0.162	0.411	28.635	22.225	47.295	21.480	16.750	31.680	**9.2e-5**
DFRIQR (Hz)	FSI	0.451	0.754	455	22	16	-0.126	-0.463	0.204	38.235	35.525	51.140	46.780	38.200	52.520	0.451
fI-slope (Hz/pA)	EXC	4.2e-8	-5.483	5356	82	92	0.417	0.281	0.544	0.123	0.068	0.178	0.056	0.031	0.094	**1.3e-7**
fI-slope (Hz/pA)	NFSI	0.053	1.934	12572.5	118	81	-0.138	-0.281	0.008	0.310	0.163	0.524	0.398	0.284	0.533	0.080
fI-slope (Hz/pA)	FSI	0.818	-0.230	391	21	16	0.041	-0.297	0.379	0.697	0.497	0.854	0.622	0.549	0.767	0.818

Differences were calculated with a two-sided Wilcoxon rank sum test and corrected for multiple comparisons (Benjamini-Hochberg).

Supplementary Table 2: Description of electrophysiological features

Feature	Definition	UMAP input
AP width	Width of the 1st AP of the rheobase sweep at half amplitude determined from threshold to peak.	Yes
AP threshold	Threshold of the first rheobase action potential determined as membrane potential at which a 10% of the peak slope of the rising phase is reached.	Yes
fast trough	Minimum membrane potential within 1.5 ms after the first rheobase AP has reached threshold level again.	Yes
slow through	Minimum membrane potential after the first rheobase AP has reached threshold level again up to the next AP or the stimulus end.	Yes
latency	Time difference between stimulus onset and threshold of the first action potential at the rheobase sweep.	Yes
rheobase rate	Number of spikes at the rheobase sweep.	Yes
peak stroke ratio	The peak stroke ratio is the peak upstroke divided by the peak down stroke	Yes
hero sweep current step	Current step of the hero sweep. The hero sweep is defined as the sweep closest to 65% of the sweep with max. firing rate	Yes
hero sweep latency	Time difference between stimulus onset and threshold of the first AP at the hero sweep.	Yes
median instantaneous rate	ISIs pooled across stimulus intensities. This is inverse to the median ISIs.	Yes
P_{90} total ISIs	90th percentile of ISIs pooled across stimulus intensities.	Yes
P_{10} total ISIs	10th percentile of ISIs pooled across stimulus intensities.	Yes
interquartile range total ISIs	Interquartile range of ISIs pooled across stimulus intensities.	Yes
adaptation ratio (last bin)	Stimulus divided into 13 bins of 77 ms each. Spike counts per bin are summed up across stimulus intensities. Ratio is calculated by first bin and last bin with non-zero value.	Yes
input resistance (highest deflection)	Slope of linear fit of IU data of the three lowest current steps. Voltage determined by membrane potential change to highest deflection within the first 200 ms of the stimulus.	Yes
input resistance (steady state)	As above but: Voltage determined by membrane potential change to steady-state potential within the last 200 ms of the stimulus.	Yes
time constant / τ	Maximum tau of hyperpolarizing current steps with a membrane deflection between 2 and 11 mV. Tau is calculated as time point when the exponential fit of the membrane potential deflection from stimulus onset to highest deflection reaches 66%.	Yes
V_m	Mean of all sweep baseline membrane potentials across all sweeps. Calculated for each sweep as mean membrane potential in the prestimulus interval.	Yes
V_m sag sweep	Baseline membrane potential at sag sweep.	Yes
delayed rectification	Ratio between steady-state membrane deflection and hypothetical steady-state deflection based on the input resistance at the most hyperpolarizing sweep.	Yes
instantaneous rectification	Ratio between highest membrane deflection and hypothetical highest deflection, based on input resistance at the most hyperpolarizing sweep.	Yes
rheobase	Current step at the rheobase sweep. Rheobase sweep is determined as the sweep with the lowest number of spikes.	Yes

sag	Difference in membrane potential between steady-state and highest deflection at the sag sweep. Sag sweep is calculated in the lowest hyperpolarizing stimulus sweep with a deflection higher than 11 mV.	Yes
sag ratio	(Sag + steady-state depolarization) divided by steady-state depolarization. Sag ratio 1 = no sag. Sag ratio 1.5 = sag is half of the steady-state depolarization.	Yes
adaptation ratio (2nd bin)	As adaptation ratio (last bin) but: ratio is calculated by first and second bin.	Yes
trough difference	Difference in membrane potential between 1st AP and 2nd last AP in the hero sweep	Yes
trough ratio	Ratio between trough difference and distance in baseline membrane potential and trough of the 2nd last AP.	Yes
CV_{ISI}	Coefficient of variation of ISIs of a sweep. Calculated as SD divided by mean at the hero sweep.	Yes
f-I slope	slope of a robust linear fit of the I-f curve.	Yes
Burst index	1-Ratio of 1st ISI divided by mean ISI of the remaining ISIs from the hero sweep.	No
AP height	Difference in membrane potential within the rheobase action potential between threshold and peak	No
peak upstroke	Maximum slope of the rising phase of the rheobase action potential	No
peak downstroke	Maximum slope of the repolarizing phase of the rheobase action potential	No
AP peak	Absolute membrane potential at the peak of the rheobase action potential	No
Maximum firing rate	Maximum firing rate from a single sweep	No

Supplementary Table3: Morphology properties of EXC and FSI

EXC feature	p_{raw}-value	z-value	ranksum	r_{rb}	r_{rb} CI1	r_{rb} CI2	LPFC median	LPFCQ1	LPFCQ3	V1 median	V1Q1	V1Q3	V1 n	LPFC n	p_{corr}-value
Total basal length	0.001	3.447	404	-0.633	-0.793	-0.408	2643.48	1959.44	3063.78	1488.83	1377.50	1848.36	11	20	0.002
Basal endings	0.028	2.195	373.5	-0.405	-0.761	0.019	24.50	21.00	26.50	18.00	15.25	26.50	11	20	0.028
Median basal length	0.004	2.869	390	-0.528	-0.733	-0.242	130.92	111.97	150.42	105.48	95.07	121.56	11	20	0.008
Total apical length	4.2e-4	3.530	406	-0.648	-0.800	-0.423	2979.41	2084.76	4152.06	1158.90	871.25	1806.68	11	20	0.001
Apical endings	2.1e-4	3.703	410	-0.680	-0.833	-0.477	24.50	20.00	29.50	15.00	13.00	17.00	11	20	0.001
Median apical length	0.002	3.076	395	-0.565	-0.770	-0.287	191.08	167.59	223.08	130.87	99.80	174.99	11	20	0.003
Soma depth	0.013	2.495	301	-0.494	-0.759	-0.162	371.47	298.03	518.72	191.02	141.70	312585.00	9	18	0.016

FSI feature	p_{raw}-value	z-value	ranksum	r_{rb}	r_{rb} CI1	r_{rb} CI2	LPFC median	LPFCQ1	LPFCQ3	V1 median	V1Q1	V1Q3	V1 n	LPFC n	p_{corr}-value
Total dendritic length	0.525	-0.636	70	0.196	-0.394	0.714	3695.19	2463.83	4177.87	3765.42	2870.87	4751.64	4	10	0.525
Median dendritic length	0.077	1.768	88	-0.510	-0.794	-0.079	175.52	154.49	187.55	126.78	92.15	158.40	4	10	0.139
Dendritic endings	0.019	-2.336	58	0.668	0.322	0.803	22.50	18.00	26.00	32.50	30.50	41.00	4	10	0.078
Total axon length	0.037	2.083	77	-0.624	-0.816	-0.269	19743.29	18324.67	23000.74	12239.43	8290.16	17166.31	4	9	0.050
Median axon length	0.007	2.700	81	-0.802	-0.819	-0.804	293.74	283.44	354.69	233.51	218.71	245.99	4	9	0.028
Axon endings	0.190	1.312	72	-0.401	-0.812	0.136	192.00	170.00	300.25	153.50	110.00	210.50	4	9	0.190

Supplementary table 3: Morphological features of EXC and FSI in LPFC and V1

Statistical differences were calculated with a two-sided Wilcoxon rank sum test and corrected for multiple comparisons (Benjamini-Hochberg). Rank-Biserial Correlation (r_{rb}) as effect size measure for the rank sum test. For FSI, the exact p-values were calculated due to the small sample size. Source data are provided as a Source Data file.

Supplementary Table 4: Mixed-model amplifier comparison and AP half-width comparison

**Linear mixed-effects model fit - Amplifier**							
**Model information:**	Observations	Fixed effects coefficient		Random effects coefficient		Covariance parameters	
	190	4		9		2	
**Formula:**	AP half-width ~ 1 + corticalArea * log(capacitance) + (1 | AmpIdx)						
**Model fit statistics:**	AIC	BIC	LogLikelihood	Deviance			
	-148.04	-128.56	80.019	-160.04			
**Fixed effects coefficients (95% CIs):**							
Name	Estimate	SE	tStat	DF	p-Value	Lower	Upper
(Intercept)	1.1768	0.0691	17.0320	186	8.188E-40	1.0405	1.3131
Cortical Area: V1	-0.3544	0.1106	-3.2057	186	0.001586	-0.5725	-0.1363
Log capacitance	0.1179	0.0293	4.0202	186	0.000084404	0.0601	0.1758
Interaction	-0.1093	0.0450	-2.4305	186	0.016027	-0.1981	-0.0206
**Random effects covariance parameters (95% CIs):**							
Name	Estimate	Lower	Upper				
Intercept Std	0.0863	0.0473	0.1576	Group AmpIdx (9 levels)			
Residual Std	0.1528	0.1379	0.1693				

**Linear Model without mixed-effects – AP half-width**							
**Model information:**	Observations	Fixed effects coefficient		Random effects coefficient		Covariance parameters	
	190	4		0		1	
**Formula:**	AP half-width ~ 1 + corticalArea * log(capacitance)						
**Model fit statistics:**	AIC	BIC	LogLikelihood	Deviance			
	-130.87	-114.63	70.43	-140.87			
**Fixed effects coefficients (95% CIs):**							
Name	Estimate	SE	tStat	DF	p-Value	Lower	Upper
(Intercept)	1.1993	0.0624	19.2250	186	4.54E-46	1.0763	1.3224
Cortical Area: V1	-0.3734	0.1192	-3.1326	186	0.002013	-0.6086	-0.1383
Log capacitance	0.1379	0.0305	4.5210	186	1.09E-05	0.0777	0.1981
Interaction	-0.1312	0.0484	-2.7117	186	0.0073223	-0.2267	-0.0358
**Random effects covariance parameters (95% CIs):**							
Name	Estimate	Lower	Upper				
Residual Std	0.1670	0.1510	0.1847	Group AmpIdx (9 levels)			

Source data are provided as a Source Data file.

Supplementary Table5: Two-way ANOVA sag ratio and R_{in}

**sag ratio**							
Source	Sum Sq.	d.f.	Mean Sq.	F	Prob>F	pEta Sq.	corrected p
Species	4.1362	1	4.13616	113.05	7.88E-24	0.192244	7.88E-24
Cell Type	2.769	2	1.38449	37.84	5.63E-16	0.137433	5.63E-16
Species:Cell Type	1.8482	2	0.92412	25.26	3.76E-11	0.096127	3.76E-11
Error	17.3789	475	0.03659				
Total	31.5512	480					
**input resistance**							
Source	Sum Sq.	d.f.	Mean Sq.	F	Prob>F	pEta Sq.	corrected p
Species	3428239	1	3428239	164.19	1.55E-32	0.254878	1.55E-32
Cell Type	2929275	2	1464637	70.15	1.88E-27	0.226172	1.88E-27
Species:Cell Type	582333.1	2	291166.6	13.94	1.30E-06	0.054913	1.30E-06
Error	10022274	480	0.03659				
Total	21892318	485					

Source data are provided as a Source Data file.
