# Supplementary material for: Intrinsic neuronal diversity as a substrate for cortical area specialization in primate vision
Source: Nat Commun. 2026 Jun 9;17:7335. doi: 10.1038/s41467-026-73734-5 (PMC13402634; doi:10.1038/s41467-026-73734-5)
Supplement: Supplementary file 2 — Reporting Summary [file 41467_2026_73734_MOESM2_ESM.pdf]

Corresponding author(s): Martinez-Trujillo J.

Last updated by author(s): Mar 20, 2026

Reporting Summary

Nature Portfolio wishes to improve the reproducibility of the work that we publish. This form provides structure for consistency and transparency in reporting. For further information on Nature Portfolio policies, see our Editorial Policies and the Editorial Policy Checklist.

Statistics

For all statistical analyses, confirm that the following items are present in the figure legend, table legend, main text, or Methods section.

n/a	Confirmed	
☐	☒	The exact sample size (*n*) for each experimental group/condition, given as a discrete number and unit of measurement
☐	☒	A statement on whether measurements were taken from distinct samples or whether the same sample was measured repeatedly
☐	☒	The statistical test(s) used AND whether they are one- or two-sided<br>*Only common tests should be described solely by name; describe more complex techniques in the Methods section.*
☐	☒	A description of all covariates tested
☐	☒	A description of any assumptions or corrections, such as tests of normality and adjustment for multiple comparisons
☐	☒	A full description of the statistical parameters including central tendency (e.g. means) or other basic estimates (e.g. regression coefficient) AND variation (e.g. standard deviation) or associated estimates of uncertainty (e.g. confidence intervals)
☐	☒	For null hypothesis testing, the test statistic (e.g. F, t, r) with confidence intervals, effect sizes, degrees of freedom and P value noted<br>*Give P values as exact values whenever suitable.*
☒	☐	For Bayesian analysis, information on the choice of priors and Markov chain Monte Carlo settings
☐	☒	For hierarchical and complex designs, identification of the appropriate level for tests and full reporting of outcomes
☐	☒	Estimates of effect sizes (e.g. Cohen's *d*, Pearson's *r*), indicating how they were calculated

Our web collection on statistics for biologists contains articles on many of the points above.

Software and code

Policy information about availability of computer code

Data collection	Raw electrophysiological data was collected with Axon pCLAMP 10&11 (Molecular Devices), Signal 5&7 (CED, Cambridge Electronic Design Limited) and Patchmaster 2x92 (HEKA Elektronik, Harvard Biosciences Inc.).<br>Imaging data was acquired with ZenBlack 2.3 (ZEISS) and LAS X (Leica).
Data analysis	Electrophysiology data were converted to NWB file format using the MATNWB repository (https://github.com/NeurodataWithoutBorders/matnwb) analyzed with custom code written in MATLAB R2023B (MathWorks). The code is publicly available on GitHub (GitHub - mfeyerab/MATFX).<br>Neuron reconstructions were done with Neurolucida 360 2021.1.3 (MBF Bioscience) and converted to the swc file format with xyz2swc (https://neuromorpho.org/xyz2swc/ui/).<br>Images in the figures are original and were created by the authors using Adobe Illustrator CS6 and CC2026.

For manuscripts utilizing custom algorithms or software that are central to the research but not yet described in published literature, software must be made available to editors and reviewers. We strongly encourage code deposition in a community repository (e.g. GitHub). See the Nature Portfolio guidelines for submitting code & software for further information.

Data

Policy information about availability of data

All manuscripts must include a data availability statement. This statement should provide the following information, where applicable:

- Accession codes, unique identifiers, or web links for publicly available datasets
- A description of any restrictions on data availability
- For clinical datasets or third party data, please ensure that the statement adheres to our policy

The marmoset data generated in this study have been deposited in the online database under primatedatabase.com. The processed marmoset data generated in this study are provided in the Supplementary Information/Source Data file. The AIBS mouse/human data used in this study are available in the AIBS database under celltypes.brain-map.org.

Research involving human participants, their data, or biological material

Policy information about studies with human participants or human data. See also policy information about sex, gender (identity/presentation), and sexual orientation and race, ethnicity and racism.

Reporting on sex and gender	*Use the terms sex (biological attribute) and gender (shaped by social and cultural circumstances) carefully in order to avoid confusing both terms. Indicate if findings apply to only one sex or gender; describe whether sex and gender were considered in study design; whether sex and/or gender was determined based on self-reporting or assigned and methods used.* *Provide in the source data disaggregated sex and gender data, where this information has been collected, and if consent has been obtained for sharing of individual-level data; provide overall numbers in this Reporting Summary. Please state if this information has not been collected.* *Report sex- and gender-based analyses where performed, justify reasons for lack of sex- and gender-based analysis.*
Reporting on race, ethnicity, or other socially relevant groupings	*Please specify the socially constructed or socially relevant categorization variable(s) used in your manuscript and explain why they were used. Please note that such variables should not be used as proxies for other socially constructed/relevant variables (for example, race or ethnicity should not be used as a proxy for socioeconomic status).* *Provide clear definitions of the relevant terms used, how they were provided (by the participants/respondents, the researchers, or third parties), and the method(s) used to classify people into the different categories (e.g. self-report, census or administrative data, social media data, etc.)* *Please provide details about how you controlled for confounding variables in your analyses.*
Population characteristics	*Describe the covariate-relevant population characteristics of the human research participants (e.g. age, genotypic information, past and current diagnosis and treatment categories). If you filled out the behavioural & social sciences study design questions and have nothing to add here, write "See above."*
Recruitment	*Describe how participants were recruited. Outline any potential self-selection bias or other biases that may be present and how these are likely to impact results.*
Ethics oversight	*Identify the organization(s) that approved the study protocol.*

Note that full information on the approval of the study protocol must also be provided in the manuscript.

Field-specific reporting

Please select the one below that is the best fit for your research. If you are not sure, read the appropriate sections before making your selection.

☒ Life sciences ☐ Behavioural & social sciences ☐ Ecological, evolutionary & environmental sciences

For a reference copy of the document with all sections, see nature.com/documents/nr-reporting-summary-flat.pdf

Life sciences study design

All studies must disclose on these points even when the disclosure is negative.

Sample size	Due to the nature of the study, i.e. post-hoc identification of cell types + multi-site collaboration, no prior calculations have been made to determine sample size. We deem our sample sizes to be sufficient to demonstrate significant divergence or lack thereof across various cell types. We assume a lack of power for FSI might conceal smaller effects particular in morphology. Biggest constraint for sample size was the yield of less numerous cell types, in particular neurons with a well-preserved morphology, which we were unable to increase in a selective manner.
Data exclusions	Electrophysiological recordings of cells were excluded entirely if the initial membrane potential was above -55 mV, input resistance or action potential waveform features could not be determined. Partial, sweep-wise exclusion of data was done by a series of specific criteria detailed in the method section. In addition, cells with 25% of electrophysiological features missing were also excluded. Exclusion criteria were not pre-established.
Replication	UMAP procedure and classification was repeated 500 times with different subsets of the dataset to increase reproducibility and validity. Our dataset is the first of its kind, so we were unable to perform our analysis on data from another source. We observed several findings (input

resistance and action potential half-width of pyramidal cells, etc.) that have been previously described in species with relative high phylogenetic proximity, i.e. macaque.

Randomization	Animals used for our study have not been randomized, but have reached a terminal endpoint for various reasons that are not related to the scientific question we pursued. Important subject data such as age, sex and health status were documented and tested as potential covariate, but showed no effect on the comparison and parameters of interest. Most important covariant for certain electrophysiological features was the recording equipment used. We consequently used a linear mixed model to verify that observed effects are due to experimental manipulations and not systematic differences caused by hardware differences.
Blinding	Blinding was not feasible nor relevant for our study. Experimenters had knowledge of the cortical area they were recording from, but cell type identification occurred after the experiment. Data analysis and data acquisition was done separately and most data was acquired by experimenters who were not involved in the analysis.

Reporting for specific materials, systems and methods

We require information from authors about some types of materials, experimental systems and methods used in many studies. Here, indicate whether each material, system or method listed is relevant to your study. If you are not sure if a list item applies to your research, read the appropriate section before selecting a response.

Materials & experimental systems

n/a	Involved in the study
☐	☒ Antibodies
☒	☐ Eukaryotic cell lines
☒	☐ Palaeontology and archaeology
☐	☒ Animals and other organisms
☒	☐ Clinical data
☒	☐ Dual use research of concern
☒	☐ Plants

Methods

n/a	Involved in the study
☒	☐ ChIP-seq
☒	☐ Flow cytometry
☒	☐ MRI-based neuroimaging

Antibodies

Antibodies used	The study used DAPI (1:4000, D1306, Invitrogen by ThermoFisher Scientific) and Alexa Fluor 633 Streptavidin (1:300, S21375, Invitrogen by ThermoFisher Scientific).
Validation	No primary antibodies were used. DAPI and Alexa Fluor 633 Streptavidin were validated against negative controls. Further validation is available at the manufacturer's website (https://www.thermofisher.com/order/catalog/product/D1306 & https://www.thermofisher.com/order/catalog/product/de/de/S21375).

Animals and other research organisms

Policy information about studies involving animals; ARRIVE guidelines recommended for reporting animal research, and Sex and Gender in Research

Laboratory animals	The study used common marmoset, Callithrix jacchus without genetic labeling.
Wild animals	The study did not involve wild animals.
Reporting on sex	The study used animals of both sex depending on the availability. Sex was not considered in the study design as there is no previous reported difference in neuron cell composition or characteristics between male and female brains. We aimed to balance data acquisition across species where it was was possible and collected cells from 33 male and 18 female monkeys. Due to the unbiased approach to patch-clamp and lack of genetic labeling in the common marmoset, the type of collected cells varied significant between each monkey. This made it not possible to collect a large enough sample size to do cell-type specific sex-based analysis. In addition females were often not available due to breeding concerns.
Field-collected samples	The study did not involve samples collected from the field.
Ethics oversight	Experiments were approved by the Animal Care Committee of the University of Western Ontario under the Canadian Council of Animal Care policy on the care and use of laboratory animals. Experiments in Germany were conducted in compliance with Directive 2021/63/EU of the European Animal Research Association and the German Animal Welfare Act as part of a study approved by the Lower Saxony State office for Consumer Protection and Food Savety (LAVES; ref. 33.19-42502-04-20/3458).

Note that full information on the approval of the study protocol must also be provided in the manuscript.

Plants

Seed stocks	*Report on the source of all seed stocks or other plant material used. If applicable, state the seed stock centre and catalogue number. If plant specimens were collected from the field, describe the collection location, date and sampling procedures.*
Novel plant genotypes	*Describe the methods by which all novel plant genotypes were produced. This includes those generated by transgenic approaches, gene editing, chemical/radiation-based mutagenesis and hybridization. For transgenic lines, describe the transformation method, the number of independent lines analyzed and the generation upon which experiments were performed. For gene-edited lines, describe the editor used, the endogenous sequence targeted for editing, the targeting guide RNA sequence (if applicable) and how the editor was applied.*
Authentication	*Describe any authentication procedures for each seed stock used or novel genotype generated. Describe any experiments used to assess the effect of a mutation and, where applicable, how potential secondary effects (e.g. second site T-DNA insertions, mosiacism, off-target gene editing) were examined.*
