# Supplementary material for: Intrinsic neuronal diversity as a substrate for cortical area specialization in primate vision
Source: Nat Commun. 2026 Jun 9;17:7335. doi: 10.1038/s41467-026-73734-5 (PMC13402634; doi:10.1038/s41467-026-73734-5)

nature portfolio

Peer Review File

Single neuron diversity supports area functional specialization along the visual cortical pathways

Corresponding Author: Professor Julio Martinez-Trujillo

This file contains all reviewer reports in order by version, followed by all author rebuttals in order by version.

Version 0:

Reviewer comments:

Reviewer #1

(Remarks to the Author)
The manuscript by Feyerabend et al., compares the properties of single neurons in a higher order cortical area, lateral prefrontal cortex (LPFC), and a primary sensory visual area, V1, in marmosets. The main findings replicate previous findings in macaque monkeys, showing that layer 2-3 excitatory pyramidal neurons in LPFC are larger, less excitable, and fire broader spikes than V1 neurons. While this dataset in marmoset is rare and valuable, there are significant concerns with regard to the novelty of the findings, the framing of the premise of the study in the context of the literature and the current state of the field, and some aspects of the methodological approaches, as detailed below.

1) The premise of the paper is at odds with the existing state of the field.

It is stated in the Abstract that “It has been assumed that each cortical area is composed of repeatable neuronal subtypes, and variations in synaptic strength and connectivity patterns underlie functional specialization” and, in the Introduction that "One issue that remains poorly investigated is whether variations in cells’ intrinsic properties and morphology could shape single neuron computations and synaptic plasticity, ultimately contributing to inter-area specialization along this axis."

On the contrary it is arguably broadly accepted that principal neurons are not composed of repeatable subtypes in the primate brain but rather that they differ markedly in different cortical regions. For many years and increasingly with the advent of the Brain Initiative Cell Census program, neuronal diversity and the effect of single neuron properties on their computations and synaptic plasticity has been a highly active field of investigation.

The premise of the paper regarding regional comparisons is misleading. The Abstract and Introduction presents the study in the context of the hierarchy of visual processing areas, i.e. how distinct areas along the primate visual stream can have different neuronal properties. The way it is framed implies that the current study investigates multiple brain areas along the visual stream in marmosets, similar to previous studies on basal dendrites in macaques (e.g., Elston & Rosa, 1997). However, the current study only compares two areas -LPFC and V1- in marmosets.

2) Many of the key findings of the paper, namely that "excitatory neurons in LPFC were larger, less excitable, and fired broader spikes than V1 neurons." are not novel and information on interneuron properties is sparse/incremental.

Much of the data presented on pyramidal neurons replicate previous studies in monkeys. Many previous studies have established that LPFC pyramidal neurons are larger than V1 pyramidal neurons in the monkey cortex (for example: Elston and Rosa, 1997; Jacobs et al., 2002; Elston, 2003; Elston, 2007; Amatrudo et al., 2012; Gilman et al., 2017) including in the marmoset (for example: Elston et al., 2001; 2006; Oga et al., 2013; Elston and Fujita, 2014). With regard to electrophysiological properties, previous studies have reported that in the rhesus monkey LPFC pyramidal neurons are less excitable, have a lower input resistance (Amatrudo et al., 2012; Gilman et al., 2016) and fire broader spikes than V1 neurons (Amatrudo et al., 2012).

Little new information is presented on interneuron properties or their differences between the two brain regions. Physiologically, C1 neurons did not differ substantially, and the notion that their axonal arbors differed is not supported by rigorous data.

3) Although most of the pyramidal neuron data replicates previous studies in primates, this is not acknowledged or discussed.

Instead, previous studies are briefly addressed as follows (Discussion):

“In primates such as the macaque monkey some studies have examined electrophysiological and morphological features of pyramidal and non-pyramidal neurons (Gonzalez-Burgos et al., 2000; Zaitsev et al., 2005, 2012; Povysheva et al., 2007, 2008; Amatrudo et al., 2012; Luebke et al., 2015; Medalla and Luebke, 2015; Gilman et al., 2017; Medalla et al., 2017). However, in general they had low sample sizes and/or were limited to one cell type and/or brain area.”

This statement is largely inaccurate. First, with regard to morphology, numerous previous studies (several of these are cited in point 2) directly compared many pyramidal neurons in multiple brain areas and species. In a comparable study to this one (because similar approaches were used), Gilman et al. (2016) characterized the detailed morphological properties of 15 V1 and 15 LPFC pyramidal neurons in the macaque while the present study characterized 4 V1 and 11 LPFC pyramidal neurons. Gilman et al., report a lower sample size for electrophysiological assessments of pyramidal neurons (n= 17 V1 and 25 LPFC) but the study was well-powered, and results were largely the same as in the present study (n= 56 V1 and 104 LPFC).

4) The proposed new classification scheme does not contribute meaningfully to the field.

The authors state that "We used the classifier to identify three classes of cells: C1 (aspiny fast-spiking interneurons), C2 (aspiny non-fast spiking interneurons), and C3 (spiny excitatory cells). We focused on C1 and C3 groups and compared neuronal intrinsic features between areas V1 and LPFC. C3 cells in V1 were more excitable, smaller, and fired narrower spikes than in LPFC. C1 neurons in the LPFC had longer latencies and a more depolarized action potential trough than V1 cells."

The classifier approach does not add to our understanding of neuronal diversity in different cortical areas in the marmoset. The existence of these broad cell types -pyramidal neurons; fast-spiking and non-fast-spiking interneurons- is very well established, and this new proposed classification/nomenclature scheme does not represent a meaningful or clarifying advance for the field.

Overall, the rationale for integrating the marmoset with mouse and human data is unclear and not discussed in the context of the literature. In the context of cell census initiatives, the aligning of marmoset with mouse and human datasets is valuable to assess inter-species differences and fine-grain features of subtypes within each broad class, which the current study does not achieve. The existence of cell diversity beyond what was already known is what the most recent cell census studies have revealed, specifically those done by the AIBS collaborators here. The current study largely ignores this body of work and does not address some important comparative questions: Are there distinct excitatory neuron/C3 subclasses in marmoset? How does excitatory neuron diversity in marmoset compare to macaques, mouse, and humans? Would clustering/classifiers based on morphological features or synaptic features yield different results? It would have been informative for instance to do re-clustering of the excitatory neuron C3 class and see if subclasses emerge. In addition, the comparative perspectives comparing marmoset to humans and mouse and especially macaques, should be highlighted in the study, given that mouse and human data are included and that the main findings related to excitatory neurons in the current study are replications of previously published rhesus monkey data.

5) Methodological Concerns and Questions

a) Sample size is a concern regarding conclusions about the morphological features of interneurons. The authors report that some inhibitory fast spiking interneurons in the LPFC had longer axons than those in V1, but this is based on only 3 V1 and 6 LPFC neurons. The authors acknowledge that assessment of axons in slices is problematic (but argue without support that cut axons are more of an issue for pyramidal neurons than for interneurons) and that "A larger sample of reconstructed cells would be needed to more systematically assess differences between areas.” The numbers of neurons used to determine morphological class are reported to be 144 in the text but add up to 134 in Figure 1C. Of these neurons, 39 were classified as interneurons- why were only 9 of these reconstructed?

b) There is ambiguity with regard to how the mouse, human and marmoset data were integrated and aligned. The UMAP clustering was based on 29 (note that in the text the number is 29 but in Figure S1A it is 21) electrophysiological parameters from all 3 species together. Then these data were split into “training” versus “test” datasets. Did the “training” and “test” datasets each contain cells from all 3 species? If so, what proportion fell into the “test” versus “training” data set in each species?

c) It is not clear how certain aspects of the clustering/classifier were implemented. For instance, in the statement “Cell type composition of training data was informed by histological data of the primate neocortex, consequently the ratio of SST, VIP and NGF cells within the training data was fixed to 2:2:1.”; what is the reference for this? Classic studies have shown that the proportion of distinct interneuron types differ across areas, specifically along the visual occipito-temporal axis (Hendry et al. 1989; Kondo et al. 1994; Glezer et al. 1998; DeFelipe et al. 1999) in macaque monkeys. Similarly, for the statement: “Marmoset and human test data set for cross-species validation was determined by unambiguous morpho-electrophysiological identity”. – what do the authors mean by “unambiguous morpho-electrophysiological identity”?

d) While experiments and data were acquired to be consistent with AIBS data, the authors noted an effect of acquisition

system on AP waveforms (Figure S2). How does this compare to the AIBS data (what acquisition system did they use)? Also, even as it appears no clustering based on acquisition system was shown, it is important that the number of V1 and LPFC cells recorded in each system is balanced and is stated in the paper.

e) Why was there no comparison of C2 type (non-fast spiking interneurons) between areas?

f) The abbreviation “hero” is not defined.

Citations

Amatrudo JM, Weaver CM, Crimins JL, Hof PR, Rosene DL, Luebke JI. 2012. Influence of highly distinctive structural properties on the excitability of pyramidal neurons in monkey visual and prefrontal cortices. J Neurosci. 32:13644–13660.

DeFelipe, J., Gonzalez-Albo, M. C., Del Rio, M. R., & Elston, G. N. (1999). Distribution and patterns of connectivity of interneurons containing calbindin, calretinin, and parvalbumin in visual areas of the occipital and temporal lobes of the macaque monkey. J Comp Neurol, 412(3), 515-526. doi:10.1002/(sici)1096-9861(19990927)412:3<515::aid-cne10>3.0.co;2-1

Elston GN, Benavides-Piccione R, Elston A, Zietsch B, DeFelipe J, Manger P, Casagrande V, Kaas JH. Specializations of the granular prefrontal cortex of primates: implications for cognitive processing. Anat Rec A Discov Mol Cell Evol Biol. 2006 Jan;288(1):26-35. doi: 10.1002/ar.a.20278. PMID: 16342214.

Elston GN, Benavides-Piccione R, DeFelipe J. The pyramidal cell in cognition: a comparative study in human and monkey. J Neurosci. 2001 Sep 1;21(17):RC163. doi: 10.1523/JNEUROSCI.21-17-j0002.2001. PMID: 11511694; PMCID: PMC6763111.

Elston GN. 2007. Evolution of the pyramidal cell in primates. In: Kaas J, Preuss T, editors. Evolution of nervous systems, Volume IV. New York: Academic Press.

Elston GN. Cortex, cognition and the cell: new insights into the pyramidal neuron and prefrontal function. Cereb Cortex. 2003 Nov;13(11):1124-38. doi:10.1093/cercor/bhg093. PMID: 14576205.

Elston GN, Rosa MG. The occipitoparietal pathway of the macaque monkey: comparison of pyramidal cell morphology in layer III of functionally related cortical visual areas. Cereb Cortex. 1997 Jul-Aug;7(5):432-52. doi: 10.1093/cercor/7.5.432. PMID: 9261573.

Elston GN, Fujita I. Pyramidal cell development: postnatal spinogenesis, dendritic growth, axon growth, and electrophysiology. Front Neuroanat. 2014 Aug 12;8:78. doi: 10.3389/fnana.2014.00078. PMID: 25161611; PMCID: PMC4130200.

Glezer, II, Hof, P. R., & Morgane, P. J. (1998). Comparative analysis of calcium-binding protein-immunoreactive neuronal populations in the auditory and visual systems of the bottlenose dolphin (Tursiops truncatus) and the macaque monkey (Macaca fascicularis). J Chem Neuroanat, 15(4), 203-237. doi:10.1016/s0891-0618(98)00022-2

Gilman JP, Medalla M, Luebke JI. Area-Specific Features of Pyramidal Neurons-a Comparative Study in Mouse and Rhesus Monkey. Cereb Cortex. 2017 Mar 1;27(3):2078-2094. doi: 10.1093/cercor/bhw062. PMID: 26965903; PMCID: PMC6059164.

Hendry, S. H. C., Jones, E. G., Emson, P. C., Lawson, D. E. M., Heizmann, C. W., & Streit, P. (1989). Two classes of cortical GABA neurons defined by differential calcium binding protein immunoreactivities. Experimental Brain Research, 76, 467-472.

Jacobs BS, Scheibel AB. (2002). Regional dendritic variation in primate cortical pyramidal cells. In: Schüz AM, Miller R, editor. Cortical Areas: Unity And Diversity. London: Taylor and Francis, Inc.

Kondo, H., Hashikawa, T., Tanaka, K., & Jones, E. G. (1994). Neurochemical gradient along the monkey occipito-temporal cortical pathway. NeuroReport, 5(5), 613-616.

Oga T, Aoi H, Sasaki T, Fujita I, Ichinohe N. Postnatal development of layer III pyramidal cells in the primary visual, inferior temporal, and prefrontal cortices of the marmoset. Front Neural Circuits. 2013 Mar 8;7:31. doi: 10.3389/fncir.2013.00031. PMID: 23483808; PMCID: PMC3592264.

Reviewer #2

(Remarks to the Author)
The current study asks if differences in hierarchical visual areas arise from their connectivity using roughly similar cell types or from intrinsic neuron properties. They use marmoset monkeys to examine two opposite ends of primate visual system, V1 and LPFC, and measure intrinsic membrane properties of patch clamped neurons plus fill them to get morphology. In

electrophysiology it is well appreciated that representations in V1 are more stable in their feature preferences over time whereas LPFC is much more plastic and involved in working memory. Thus, the possibility that some of these differences in function could be supported by differences in neuron properties is an exciting question. By contrast there is not much support for such difference between neuron properties in mice (which have been more extensively studied using these methods), and if primates do exhibit strong differences it could underlie an important part of species difference and the greater cognitive flexibility achieved in primate behavior. The work presented is important and the methods applied and resulting data are valuable and of high quality.

The results of this study first establish a basic set of cell types (three broad classes, fast spiking, non-fast spiking non-spiny neurons, and excitatory spiny neurons) and show how they compare to former studies in mice and humans. Then they examine differences between V1 and LPFC in the marmoset and find several significant and interesting differences: 1) LPFC excitatory neurons have a higher threshold to fire (correspondingly lower mean rates), 2) LPRC fast spiking cells has longer latencies to fire, 3) LPFC excitatory and fast spiking neurons were more likely to have burst firing. These intrinsic firing differences could support some differences in function between V1 and LPFC in regards to plasticity of representations and working memory. Overall I found the manuscript well written with clear illustrations of data.

I have only minor comments for clarity to improve the manuscript:
- In the abstract, the emphasis so much on the visual system as opposed to sensory vs frontal/pre-frontal cortical differences. It seems vision is not critical to the current study but rather comparing a sensory vs pre-frontal area. ALSO, in the abstract, IT SHOULD BE NOTED THE STUDY FOCUSED ON Layers 1-3 of cortex predominantly sampling from them.
- Introduction: no comments
- Methods: no comments
- Results: On Fig 2C, A) the marmoset/human cells seem to cluster in different parts of the space than the total UMAP clusters which mainly are driven by mouse data). Is this difference significant? This might just be that there are so many mouse (gray points in Fig 2C upper left) that it appears like a uniform covered area whereas the density in mice is actually comparable to that in human/marmoset showing more sampling in the central region of the main UMAP cluster rather than the edges. What would be the implications if this is a real distribution difference (less diversity in marmoset and human types?).
- Results, still Figure 2C: For the SAG difference mentioned in text, is the difference statistically significant and how was it tested (stat not included though difference looks clearly significant visually). Can more be said briefly about how a higher SAG effects neuron firing … it would lead to lower or higher firing rates, to more or less spike rate adaptation?
- Results Figure 3, it should be stated more clearly what inputs were used to train the classifier (presumably the ephys data from Fig 2C, AP width, latency, SAG ratio?) but not morphology data fom Fig 2D?). This could go in the first paragraph of this section where forest classification is first described. What is shown in Figure 2E is also unclear and needs better explanation.
- Results in Fig 4, could the authors provide a brief sentence to describe the impact in terms of firing in neurons for input resistance, rheobase, and rectification? It is only mentioned in the text that this would mean LPFC cells are less excitable, but I suspect this would also change their membrane time constants for integration (i.e., are LPFC cells slower and more sluggish in their firing, slower time constants). Also the text mentions spike rate adaptation as larger in LPFC … does that derive from these parameters, perhaps from the higher rheobase (threshold for action potential generation). What is the “herosweep” and how would it effect firing? Some brief definition of terms would be useful for a more general audience.
- Results for Fig 6 seems clear, only wish to change the shading in 6C and 6D to better show when the differences are significant (maybe use 2 std for the shaded areas rather than full range?). Also it should be better indicated which of those differences are significant.
- In discussion, paragraph 1, it is unclear what this phrase, “a more depolarized action potential trough” means and seems to introduce a result that I must have missed in the results section … perhaps this finding should be emphasized better in the text of results. I take it that a different action potential shape (less negative deflection in the trough) was seen for the fast-spiking C1 cells in LPFC than those in V1 … are there any implications for their function that they have this property?.
- In Figure 2, the UMAP structure identifies fast-spiking (narrow AP) and excitatory (broad AP) as distinct clusters with an intermediate zone that includes non-fast spiking interneurons (VIP or SST) (would be intermediate spiking types). Questions: it is mentioned in text that AP width mainly drives the horizontal axis in the UMAP cluster plot, but is there any similar intuition for what drives the vertical axis in that plot? Also, it should be mentioned what a higher SAG ratio would mean in terms for firing in neurons.
- In Figure 4F, the difference seem to be circularly defined? I suppose that one thing to clarify is that burst or not-burst is defined by an intra-cellular test to evoke a burst, but then the burst index I assume is defined using a different index from firing. Can that be clarified?
- In Figure 5, can it be briefly explained what is the “hero rheobase” and what it measures?

Reviewer #3

(Remarks to the Author)
The manuscript "Single neuron diversity supports area functional specialization along the visual cortical pathways" by Feyerabend et al. is a multi-center effort that characterizes the morphological and electrophysiological properties of >140 single cells in marmoset V1 and prefrontal cortex (PFC) and compared them to mouse and human cell type properties. The study quantifies that PFC neurons are larger, less excitable and fired broader spikes an V1 cells, that subgroups of marmoset interneurons have long axons and slower response latencies, and that burst firing was more common in PFC excitatory and inhibitory cells. These area- and species- specific findings are described in the context of an impressive overall commonality of the distinct features of fast spiking versus spiny principal cells.

The study reaches these conclusions with state-of-art techniques, a comprehensive cell characterization protocol, advanced clustering approaches and fair statistical evaluations. The writing is concise, and the conclusion are well justified.

Overall, this is an impressive study that provides an important characterization of cell types in visual and prefrontal cortex of marmosets, clarifying their differences to cells types characterized in the mouse and discussing apparent unique characteristics of some cell types.

The study has the potential to become a key reference for future studies aiming at understanding cell type specific functions in V1 and LPFC of the marmoset. The results are critical for interpreting the functional specializations of the visual and prefrontal cortex in primate brains. The provided results may prove important for understanding cell type specific alterations in psychiatric 'prefrontal cortex' disorders.

There were only few aspects of the study that deserve consideration - mostly to probe for a comprehensive description of the dataset.

- The results of this study are impressive as they reveal commonalities but also important differences of cell characteristics between species and brain areas. The authors frame the overall results in terms of reflecting "neuron diversity" and supporting "area specialization" (see title of the paper) in the "visual cortical pathways" (somehow assuming the LPFC is part of the visual pathway?). However, neither the diversity nor the functional specialization are particularly novel. Guy Elston and others have multiple contribution that clarified the (PFC) area specificity and this work could be cited as it seems remarkable consistent with the marmoset findings and difference of human and mice cells are reported in previous studies. It would help a reader to see in the title and abstract some specific result that this work uniquely points (maybe that increased diversity of interneuron features (subtypes with longer axons? NGF subtypes,...), bursting, or excitability) differences point to a functional specializations of the PFC in the primate lineage ?).

- It is not clear why the characterization of the interneuron types assigned to class 2 is missing. Readers are exposed to neuron types that are assigned to a class 2, but do not receive information on how variable the class 2 cells are. This information is missing but would help a reader to discern how different (on average) C2 class neuron properties are to the C1 and C3 class. The authors may consider a supplementary results section that briefly describe what the e-types 1, 2-3, and 4 are that characterize class 2 neuron types, and add a table about electrophysiological or other properties of cells in that class.

- Classification of the cell group labels (C1,2,3) used the UMAP components (the results text says so) or a subset of PCAs of the raw features (the methods describe this). It is not made clear why these decisions were made, as the classification would be closer to the actual cell properties if the classifier used the morphological and electrophysiological features instead of the UMAP / PCA components. These components are already describing the most distinguishable features so that a classifier may have high label assignment accuracies because the input already pre-clustered them.
A related question is why the authors decided on using a 2 and 3 UMAP component visualization instead of performing unsupervised clustering of the data and testing how consistent marmoset cell labels are assigned to the clustered classes. It is not apparent what the advantage is - if any - of the chosen approach.
It would enhance the appeal of the study if a clustering and cluster visualization approach is used that allows a direct comparison to the relevant literature of cell types (in mice or monkeys), or explicitly discuss the rationale for choosing the UMAP approach (mostly for visualization?).

- Figure 6 is supposed to compare V1 and LPFC fast spiking neurons and pyramidal cells, but the figure only allows appreciating the differences between brain areas while the cell type differences are difficult to see because the (y-limit) scales for pyramidal cells and fast spiking cells in C versus D are different. it would help to see panels (e.g. as new E and F) showing fast spiking and pyramidal cells in the same plot separately for V1 and LPFC ?

- Why does Figure 6 show the range but not the standard deviation or standard error of the morphological features?

- p. 7 what are the "e-type[s] ranging from Inh_4 to Inh_7" and what are "e-type 2 or 3 " ? Are there e types that uniquely characterize SST and VIP neurons? How similar are the distributions of these e-Types in the marmoset and mouse datasets?

minor
- please add y-label for figure 3F
- Figure 3D "prediction"
- p 14 "trains of spikes " to "train .. "
- reference Froudist-Walsh et al., 2023 is missing in the bibliography.

Version 1:

Reviewer comments:

Reviewer #1

(Remarks to the Author)
The authors were highly responsive to the reviews of their paper. Initial concerns about the novelty of the work and some methodological questions have each been comprehensively addressed and are alleviated. The revised manuscript is of excellent quality and represents a significant contribution to the field.

Reviewer #2

(Remarks to the Author)
The authors responses have addressed all of my comments. I find the revised manuscript to be much improved, including better references to the previous literature to address Reviewer #1's concerns as well as better acknowledging limitations of their study. I do not have any further concerns to address for this manuscript.

Reviewer #3

(Remarks to the Author)
The revisions positively and constructively addressed all comments on the original paper. These were important clarifications and overall impressive revisions.

Open Access This Peer Review File is licensed under a Creative Commons Attribution 4.0 International License, which permits use, sharing, adaptation, distribution and reproduction in any medium or format, as long as you give appropriate credit to the original author(s) and the source, provide a link to the Creative Commons license, and indicate if changes were made.
In cases where reviewers are anonymous, credit should be given to 'Anonymous Referee' and the source.
The images or other third party material in this Peer Review File are included in the article’s Creative Commons license, unless indicated otherwise in a credit line to the material. If material is not included in the article’s Creative Commons license and your intended use is not permitted by statutory regulation or exceeds the permitted use, you will need to obtain permission directly from the copyright holder.
To view a copy of this license, visit https://creativecommons.org/licenses/by/4.0/

Answers to the reviewers' comments

We thank you for taking the time to consider our manuscript and for your patience while waiting for this revision. We have substantially revised the paper and addressed all reviewer comments. Reviewers 2 and 3 found our findings significant and novel. Reviewer 1 expressed concerns about the novelty, suggesting that the results mostly replicate some previous macaque studies. We respectfully take a different view. While our findings for pyramidal neurons share some similarities with prior work in macaques, our study was conducted in marmosets (a different species) and examined both pyramidal cells and interneurons. In addition, we report novel observations within pyramidal neurons themselves, such as differences between V1 and LPFC in (i) burst properties, (ii) sag and (iii) changes in the excitability ratio between pyramidal cells and fast-spiking interneurons. To the best of our knowledge, these aspects have not been previously reported. Given the evolutionary divergence between New World and Old World monkeys, direct generalization from macaques to marmosets cannot be made. Thus, our results provide new insights into cortical microcircuit function in primates. Importantly, all reviewers provided constructive suggestions that helped to improve the manuscript.

The reviewer comments have prompted us to conduct new experiments, add more animals and neurons to our sample, and refine our claims and interpretation of the data. Increasing our sample size also allowed us to refine and expand our results on both inhibitory interneurons and pyramidal cells. We have added an author to the paper who participated in collecting the new data (Khatibi VA). We highly appreciate the opportunity to revise the manuscript. Our point-by-point answers are below.

Reviewer 1

General comment:
The manuscript by Feyerabend et al., compares the properties of single neurons in a higher order cortical area, the lateral prefrontal cortex (LPFC), and a primary sensory visual area, V1, in marmosets. The main findings replicate previous findings in macaque monkeys, showing that layer 2-3 excitatory pyramidal neurons in LPFC are larger, less excitable, and fire broader spikes than V1 neurons. While this dataset in marmoset is rare and valuable, there are significant concerns with regard to the novelty of the findings, the framing of the premise of the study in the context of the literature and the current state of the field, and some aspects of the methodological approaches, as detailed below.

We thank the reviewer for this critical feedback. We realize that our original manuscript did not sufficiently emphasize the evolutionary distinction between New World (marmoset) and Old World (macaque) primates, nor did it clearly separate our novel interneuron findings from the pyramidal cell data. We appreciate the reviewer prompting us to clarify this. We have substantially revised the Introduction and Discussion to explicitly contextualize the value of comparative primate studies and have added significant new data to further distinguish our findings from previous macaque literature. The reviewer acknowledges that the dataset we provide is valuable. Indeed, our study is the first to compare the electrophysiological features of both excitatory and inhibitory neurons between areas V1 and LPFC in marmosets, and provide a database resource similar to the one that the Allen Institute has provided for mice and humans (AIBS database). Marmosets are New World monkeys that split from Old World monkeys ~45 million years ago. While previous work in macaques (e.g., Gilman et al. 2017) provides an important foundation, we argue that data from marmosets are critical for understanding the evolutionary conservation or divergence of intrinsic neuronal features across primate species. As the reviewer notes, electrophysiological datasets for this species are rare, and we have revised the text to better articulate why testing across primate species is necessary for the field (see Introduction and Discussion in the new version of the manuscript). Below are specific answers to the reviewers' concerns, details of new data we have collected and incorporated into the manuscript and changes we have made across the sections to clarify the contributions and novelty of our study.

1) It is stated in the Abstract that “It has been assumed that each cortical area is composed of repeatable neuronal subtypes, and variations in synaptic strength and connectivity patterns underlie functional specialization” and, in the Introduction that "One issue that remains poorly investigated is whether variations in cells’ intrinsic properties and morphology could shape single neuron computations and synaptic plasticity, ultimately contributing to inter-area specialization along this axis.” On the contrary it is arguably broadly accepted that principal neurons are not composed of repeatable subtypes in the primate brain but rather that they differ markedly in different cortical regions. For many years and increasingly with the advent of the Brain Initiative Cell Census program, neuronal diversity and the effect of single neuron properties on their computations and synaptic plasticity has been a highly active field of investigation.

We thank the reviewer for these detailed comments. In no way have we attempted to disregard the work of the Brain Initiative investigators; in fact, the present work was conducted under the umbrella of NEURONEX, which was part of the Brain Initiative. The reviewer comments have motivated us to collect additional data, conduct further analyses, and revise the manuscript text and findings. Now we have added more animals and more neurons to our database and analyses. We have also rewritten the Abstract, Introduction and Discussion, highlighting the novelty of our work.

The reviewer argues that the principle of serial homology in the neocortex has been disproved in primates, and we shall not motivate our study that way. We do acknowledge that a study in macaques has shown that principal/pyramidal neurons vary in electrophysiological features between V1 and LPFC (Gilman et al. 2017). However, no electrophysiological study in primates has shown that the same applies to interneurons. We list studies on the morphology of pyramidal cells below. In the new version of the paper, we place less weight on the argument of serial homology and focus on comparing the three main cell classes’ (EXC, NFSI, and FSI) e-features between areas V1 and LPFC of the marmoset. We still discuss the argument in the Discussion section, taking into account the reviewer’s suggestions (see section ‘Serial homology and neuronal diversity in the primate visual system’ of the Discussion). We consider this to be important.

Regarding anatomical studies, we carefully examined this issue. Below is a list of anatomical studies in the marmoset, along with comments summarizing the main differences with our study.

Elston GN, Tweedale R, Rosa MG. Cellular heterogeneity in cerebral cortex: a study of the morphology of pyramidal neurones in visual areas of the marmoset monkey. J Comp Neurol. 1999 Dec 6;415(1):33-51. (*focuses on pyramidal neuron morphology, especially spines on basal dendrites, in the marmoset*)

Elston GN, Rosa MG, Calford MB. Comparison of dendritic fields of layer III pyramidal neurons in striate and extrastriate visual areas of the marmoset: a Lucifer yellow intracellular injection. Cereb Cortex. 1996 Nov-Dec;6(6):807-13. (*focuses on pyramidal neuron morphology, especially spines on basal dendrites*)

Elston GN, Rosa MG. Ipsilateral corticocortical projections to the primary and middle temporal visual areas of the primate cerebral cortex: area-specific variations in the morphology of connectionally identified pyramidal cells. Eur J Neurosci. 2006 Jun;23(12):3337-45. (*focuses on pyramidal neuron morphology*)

Elston GN, Benavides-Piccione R, Elston A, Zietsch B, Defelipe J, Manger P, Casagrande V, Kaas JH. Specializations of the granular prefrontal cortex of primates: implications for cognitive processing. Anat Rec A Discov Mol Cell Evol Biol. 2006 Jan;288(1):26-35. This paper concludes: “We propose that 1. circuitry in V1, V2, and gPFC within any given species differs in its functional capabilities and 2. There are dramatic differences in the functional capabilities of gPFC circuitry in different species, which are central to the different cognitive styles of primates. In particular, the highly branched, spinous neurons in the human gPFC may be a key component of human intelligence.” (*The paper focuses on the*

morphology of pyramidal neurons and dendritic spine count. It does not examine interneurons nor e-features).

Elston GN, Benavides-Piccione R, DeFelipe J. The pyramidal cell in cognition: a comparative study in human and monkey. J Neurosci. 2001 Sep 1;21(17):RC163. (*focuses on pyramidal neuron morphology*).

Oga T, Aoi H, Sasaki T, Fujita I, Ichinohe N. Postnatal development of layer III pyramidal cells in the primary visual, inferior temporal, and prefrontal cortices of the marmoset. Front Neural Circuits. 2013 Mar 8;7:31. (*Focuses on pyramidal cells anatomy, not electrophysiology. It does not explore PFC area 46 but area 12*).

Now we cite these studies in the Introduction and Discussion sections. However, as mentioned above, we have found no studies comparing the e-features of inhibitory interneurons and their relationship to morphology across cortical areas in primates. Our study is the first to do so using a large sample of neurons in marmosets. Regarding comparisons of principal/pyramidal cells' e-features across cortical areas, one study in macaques found that some e-features differ between V1 and LPFC (Gilman et al. 2017), and another study also in macaques between LIP and LPFC (Gonzalez-Burgos et al. 2019). We cite those studies in the manuscript and clarify their contributions. Below, we expand on the differences between Gilman et al. (2017) and our study, as well as the novel aspects of our study.

Gilman JP, Medalla M, Luebke JI. Area-Specific Features of Pyramidal Neurons: A Comparative Study in Mouse and Rhesus Monkey. Cereb Cortex. 2017 Mar 1;27(3):2078-2094.

Here are important differences with our study in species, sample size, and cell type comparisons:

- Gilman's study used 12 adult **macaques** (42 pyramidal cells, 17 in V1 and 25 in LPFC); we used 51 adult **marmosets** (483 neurons, 214 in V1 and 269 in the LPFC). (See first paragraph of the Results section).
- Gilman et al. study is restricted to pyramidal cells. We focus on both pyramidal cells and interneurons, which account for more than half of our sample (see Results Figs. 1-3).

Novel aspects of our study relative to Gilman et al. study are:

- The species (common marmoset); our study is the first to explore e-features of neurons in V1 and LPFC of marmosets.
- Interneurons (our study is the first to explore and report differences in the e-features and morphology of interneurons between area V1 and LPFC in any primate species).
- Pyramidal cells: we still report differences in pyramidal cells' e-features that were not reported by the Gilman et al. study (e.g., differences in bursting between V1 and LPFC layers II/III neurons).
- Our study also reports differences in sag between areas V1 and LPFC in all neuronal classes.
- The classifier analyses: our study provides a method for classifying neuronal types (in the absence of genetic pre-labeling) into three main categories that generalize across mice, humans and marmosets.
- The resource we have created: our study has developed a unique resource of cell types, e-features, and morphology in marmosets, using the same protocol and methods as the AIBS/BICCN census study for mice and humans. This will facilitate modeling and comparative studies (see https://primatedatabase.com).

2) The Abstract and Introduction presents the study in the context of the hierarchy of visual processing areas, i.e., how distinct areas along the primate visual stream can have different neuronal properties. The way it is framed implies that the current study investigates multiple brain areas along the visual

stream in marmosets, similar to previous studies on basal dendrites in macaques (e.g., Elston & Rosa, 1997). However, the current study only compares two areas - LPFC and V1 - in marmosets.

The reviewer's main argument is that we sample only two areas along the hierarchy of visual processing, yet we imply that we sample more areas. It was not our intention to confuse readers. As the reviewer correctly says we conduct regional comparisons between V1 and LPFC. To correct this issue we have modified the title "**Intrinsic Neuronal Diversity as a Substrate for Cortical Area Specialization in Primate Vision**". We focused on V1 and LPFC because these areas are situated at the extremes of the cortical processing hierarchy, and there is at least one study on macaque pyramidal cells (Gilman et al., 2017) that we can compare with our marmoset study. Without diminishing the value of the anatomical studies the reviewer refers to, they are not comparable to our study. In such studies, the specimen can be kept for days or months after perfusion, and one can make optimal use of a single animal by sampling several areas. In our study, we made an extraordinary effort to have several scientists conduct patch-clamp experiments as animals became available. Perfusion (ACSF to flush out the blood) is undertaken quickly, and while the tissue is viable (usually for 24 hours) we perform recordings. We usually obtain ~10-15 recordings per animal, which also varies across animals. We recorded at two sites: Canada (Western University) and Germany (The University of Goettingen) (see Methods section). Our sample size is 51 animals, the largest in any primate study and the only sample of its kind in marmosets. Moreover, the collected data had to undergo quality control, which is not trivial, and we lost cells during these strict controls because the recordings or samples were (just) not optimal. Finally, while patching non-labelled pyramidal cells is relatively straightforward due to their characteristic morphology, patching non-labelled interneurons is more difficult. We were able to do so in our study, thanks to our accumulated expertise and the joint effort and coordination between the different laboratories.

3) Many of the key findings of the paper, namely that "excitatory neurons in LPFC were larger, less excitable, and fired broader spikes than V1 neurons." are not novel and information on interneuron properties is sparse/incremental. Much of the data presented on pyramidal neurons replicate previous studies in monkeys. Many previous studies have established that LPFC pyramidal neurons are larger than V1 pyramidal neurons in the monkey cortex (for example: Elston and Rosa, 1997; Jacobs et al., 2002; Elston, 2003; Elston, 2007; Amatrudo et al., 2012; Gilman et al., 2017) including in the marmoset (for example: Elston et al., 2001; 2006; Oga et al., 2013; Elston and Fujita, 2014). With regard to electrophysiological properties, previous studies have reported that in the rhesus monkey LPFC pyramidal neurons are less excitable, have a lower input resistance (Amatrudo et al., 2012; Gilman et al., 2016) and fire broader spikes than V1 neurons (Amatrudo et al., 2012). Little new information is presented on interneuron properties or their differences between the two brain regions. Physiologically, C1 neurons did not differ substantially, and the notion that their axonal arbors differed is not supported by rigorous data.

We thank the reviewer for this critical feedback. Our original manuscript did not sufficiently emphasize the evolutionary distinction between New World (marmoset) and Old World (macaque) primates, nor did it clearly separate our novel interneuron findings from the pyramidal cell data. We have substantially revised the Introduction and Discussion to explicitly contextualize the value and novelty of our study relative to previous ones (see answer to previous comment). One cannot directly extrapolate the results of electrophysiological studies in macaques to marmosets (a New World primate with a less developed LPFC, that split from Old World primates ~45 million years ago). We report differences in bursting in pyramidal neurons (not reported in existing macaque study) and an inversion of the EXC to FSI excitability relationship between V1 and LPFC that was not reported and cannot be predicted by previous studies. Our study, to our knowledge, is the first to comprehensively compare e-properties of excitatory and inhibitory neurons across an early and a late area of the primate visual processing hierarchy. We report changes in the e-features of interneurons, particularly of FSI, with less pronounced/straightforward changes in NFSI.

Regarding anatomical properties, the reviewer's comment has motivated us to increase our sample size (see comment below) and revise this section of the manuscript (see new **Fig. 6**). We have also modified the classification into three classes and labeled the different groups of neurons according to the standard grouping of cell types in the neocortex (see new **Fig. 3**). We should clarify that our goal was not to introduce a new classification scheme but to compare the properties of pyramidal cells and the main categories of interneurons between areas V1 and LPFC of the marmoset neocortex. See also the answer to comment 1 for a summary of the differences between our study and the studies the reviewer cites in the comment. All these studies have been added to the citations list and are referred to when appropriate in the Introduction and Discussion sections.

4) Previous (pyramidal) studies in primates, are not acknowledged or discussed properly.

"In primates such as the macaque monkey some studies have examined electrophysiological and morphological features of pyramidal and non-pyramidal neurons (Gonzalez-Burgos et al., 2000; Zaitsev et al., 2005, 2012; Povysheva et al., 2007, 2008; Amatrudo et al., 2012; Luebke et al., 2015; Medalla and Luebke, 2015; Gilman et al., 2017; Medalla et al., 2017). However, in general they had low sample sizes and/or were limited to one cell type and/or brain area."

This statement is largely inaccurate. First, with regard to morphology, numerous previous studies (several of these are cited in point 2) directly compared many pyramidal neurons in multiple brain areas and species. In a comparable study to this one (because similar approaches were used), Gilman et al. (2016) characterized the detailed morphological properties of 15 V1 and 15 LPFC pyramidal neurons in the macaque while the present study characterized 4 V1 and 11 LPFC pyramidal neurons. Gilman et al., report a lower sample size for electrophysiological assessments of pyramidal neurons (n= 17 V1 and 25 LPFC) but the study was well-powered, and results were largely the same as in the present study (n= 56 V1 and 104 LPFC).

We thank the reviewer for pointing this out, and we apologize for omitting some citations. In particular, anatomical and transcriptomic studies were not adequately cited due to our focus on electrophysiology. We went through a list of studies in macaque monkeys and marmosets at the reviewers' suggestion, and we have added the citations to the manuscript. Some of these studies are summarized in the answer to comment 1.

Regarding sample size, the study of Gilman et al., the only comparison between V1 and LPFC pyramidal cell e-features, uses 17 V1 neurons and 25 LPFC cells, a total of 42 pyramidal cells in 12 macaques. Our study recorded from 269 cells in LPFC and 214 in V1 in 51 marmosets. The sample size in our study is thus substantially larger, at least from an electrophysiological point of view. Our rationale here is that reported differences in e-features may depend on the power (sample size) of the study to detect significant differences. Additionally, as we sample more neurons, we are more likely to record from less frequent cell types that can be missed when recording from fewer neurons. In a field where the diversity of neuronal types remains an open question, it is essential to have large sample sizes to capture variability. What constitutes a (sufficiently) large sample size is debatable. Here we refer to our sample size relative to one of the Gilman study.

Power: This is a complicated subject; power is interpreted in relation to the anticipated meaningful differences in the variable of study. For example, Gilman et al. 2017, did not describe the differences in bursting between pyramidal cells in V1 and LPFC. This could be interpreted as species-specific or as due to their lower sample size relative to ours. Gilman et al. did not sample interneurons.

Regarding morphology, the reviewer is correct that our sample size was very limited. We therefore collected additional morphological data, which we now incorporate into the paper. Pyramidal V1=11, LPFC=20; Fast spiking interneurons V1=4 LPFC=10, for a total of 45 reconstructions. In part, it has taken us a long time to answer the reviewers' comments because we were reconstructing neurons. This is an ongoing project, but with the current sample sizes, we observed quantitative differences in morphology. It is possible that we did not detect some other existing differences because of the sample

size. We acknowledge that it is still moderate. We have also introduced new analyses that relate morphological features to e-features, yielding a novel and intriguing finding (current Fig. 4 B and E). We thank the reviewer for prompting us to think more deeply and to undertake additional work to address the concerns.

5) The proposed new classification scheme does not contribute meaningfully to the field. The classifier approach does not add to our understanding of neuronal diversity in different cortical areas in the marmoset. The existence of these broad cell types -pyramidal neurons; fast-spiking and non-fast-spiking interneurons- is very well established, and this new proposed classification/nomenclature scheme does not represent a meaningful or clarifying advance for the field.

We appreciate the reviewer's comment. However, we believe there is a misunderstanding. The purpose of the classifier is not to explore neuronal cell type diversity in the marmoset. The purpose was to segregate the main cell types (EXC, FSI, and NFSI), so we could compare the e-features between areas V1 and LPFC located at the extremes of the visual processing hierarchy. We used the classifier because we do not have genetic labels, and we needed to find a reliable method to segregate the three main classes of neurons. This is relevant because these three main classes have different functional roles in the computations underlying visual processing. To make this clear, we have better clarified this rationale in the manuscript (see third paragraph of the Introduction and third paragraph of the 'Classifying neuron types using e-features' section in the Discussion), and now refer to the classes according to the main cell types described based on electrophysiological/functional properties (EXC, FSI, and NFSI). Finally, our classifier provides a method to segregate these main cell types (e.g. in the absence of genetic labels) in marmoset studies, which generalizes to humans (see **SFig. 4**). This offers a means to studies that do not use genetic labels to identify cell types during electrophysiological recordings (e.g., slice studies in non-human primates and humans or in other species). We have also made our data publicly available, which facilitates future studies on this topic using more sophisticated informatics tools (see https://primatedatabase.com).

6) Overall, the rationale for integrating the marmoset with mouse and human data is unclear and not discussed in the context of the literature. In the context of cell census initiatives, the aligning of marmoset with mouse and human datasets is valuable to assess inter-species differences and fine-grain features of subtypes within each broad class, which the current study does not achieve. The existence of cell diversity beyond what was already known is what the most recent cell census studies have revealed, specifically those done by the AIBS collaborators here. The current study largely ignores this body of work and does not address some important comparative questions: Are there distinct excitatory neuron/C3 subclasses in marmoset? How does excitatory neuron diversity in marmoset compare to macaques, mouse, and humans? Would clustering/classifiers based on morphological features or synaptic features yield different results? It would have been informative for instance to do re-clustering of the excitatory neuron C3 class and see if subclasses emerge. In addition, the comparative perspectives comparing marmoset to humans and mouse and especially macaques, should be highlighted in the study, given that mouse and human data are included and that the main findings related to excitatory neurons in the current study are replications of previously published rhesus monkey data.

We appreciate this comment; however, fully addressing the reviewer's points would require a separate study that would deviate from our goals in the present study. Such studies of cell types require larger sample sizes to map their diversity and when ever possible incorporate transcriptomic methods. We did think about conducting an interspecies comparison; however, the existing data in mice are mainly in V1, whereas in humans, it is the temporal cortex and a few other areas that depend on the availability of patient tissue, but not V1. In our study, we have shown that electrophysiological and anatomical features differ between V1 and LPFC, even though cells still retain general features of the three main classes we characterize, which are widely accepted as general categories. We argue that to properly address the

reviewer requests, one must have data from homologous areas across the different species. To our knowledge, these data do not exist for visual areas other than V1. We believe it is important to first establish variability between areas (this study) and then explore subcategories with reasonably large sample sizes per area across other species. This is our goal over the next few years. We have already started to collect a dataset from macaques that we are further expanding and exploring. We are working on a separate manuscript to address this issue.

7) The authors report that some inhibitory fast spiking interneurons in the LPFC had longer axons than those in V1, but this is based on only 3 V1 and 6 LPFC neurons. The authors acknowledge that assessment of axons in slices is problematic (but argue without support that cut axons are more of an issue for pyramidal neurons than for interneurons) and that "A larger sample of reconstructed cells would be needed to more systematically assess differences between areas." The numbers of neurons used to determine morphological class are reported to be 144 in the text but add up to 134 in Figure 1C. Of these neurons, 39 were classified as interneurons- why were only 9 of these reconstructed?

The reviewer is correct. We have considered this comment and increased the sample size by adding new reconstructed cells (see **Fig. 6**). This has been a significant amount of work, and we have done as much as we could with the resources available to us. This is an ongoing project that may take years to complete, depending on whether reliable methods for automatic reconstruction become more widely available. In the current manuscript, we have reached a sample size sufficient to draw conclusions, and we have done so. The lower number of reconstructed cells (FSI) relative to the number of cells we recorded is due to: (i) that we recorded from so many more NFSI than FSI neurons, (ii) the filling quality of FSI neurons had to be sufficient to draw meaningful quantitative data out of the reconstructions and (iii) the intensive labor required to reconstruct a single neuron, mainly due to the axon (~80+ hours per neuron). We have corrected the issue the reviewer points out in the text and **Fig. 1B**. We determined the dendritic type for a total of 212 neurons. Out of the 85 aspiny neurons (81 after eliminating morphologies with missing e-features; see **UMAP-Methods** and **Fig 3E**), we reconstructed 14 FSI and 19 NFSI.

8) In the abstract, the emphasis so much on the visual system as opposed to sensory vs frontal/pre-frontal cortical differences. It seems vision is not critical to the current study but rather comparing a sensory vs pre-frontal area. ALSO, in the abstract, IT SHOULD BE NOTED THE STUDY FOCUSED ON Layers 1-3 of cortex predominantly sampling from them.

Thank you for the comment regarding layers 1-3. We fully acknowledge that, and now we clarify it in the Abstract and several other sections of the manuscript. We focus on these layers due to their importance in visual and cognitive computations, their expansion in primate LPFC, and the sample size we could achieve in our study. Regarding generalizing to other areas, we do not feel comfortable with that generalization. Vision is particularly developed in primates, and there is no reason to assume the features we find in the visual system will be equally present in other sensory systems. In fact, the amount of neocortex dedicated to vision is larger than that dedicated to any other sensory modality in the marmoset and most diurnal primates. Some systems, such as the olfactory system, regressed when primates invaded the diurnal niche and began using vision to detect food sources and predators at long distances (see Kaas 2022 on “how primates escaped the nocturnal bottleneck,” and Martinez-Trujillo TICS 2025). Epigenetic changes across the lifespan induced by the use of certain sensory modalities may affect neuronal diversity and e-features differently in different sensory systems. Since we did not explore areas such as SI or A1, we again do not feel comfortable extending the Discussion to other sensory systems.

9) There is ambiguity with regard to how the mouse, human and marmoset data were integrated and aligned. The UMAP clustering was based on 29 (note that in the text the number is 29 but in Figure S1A it is 21) electrophysiological parameters from all 3 species together. Then these data were split into

“training” versus “test” datasets. Did the “training” and “test” datasets each contain cells from all 3 species? If so, what proportion fell into the “test” versus “training” data set in each species?

Thank you for the opportunity to clarify our methodology regarding the crucial integration and alignment of mouse, human, and marmoset data. First, we apologize for the ambiguity in the original text and the discrepancy between the main text and **SFig. 1A**. The correct number of e-features used for UMAP clustering was 29. We reorganized **SFig. 1** and corrected that mistake.
Second, to directly address your question: We used only mouse neurons for the training dataset. The training dataset has 1191 cells consisting of 60% EXC, 25% NFSI and 15 % FSI for each iteration together with a test dataset of 150 cells of the same composition (see **Fig. 3A**). In addition, we made a random selection for each species (mouse, marmoset and human) from a pool of identified cells the classifier had never seen. The test datasets only contained cells from one species. The marmoset test dataset contained 70 cells with 57% EXC, 29% NFSI and 14% FSI and the human test dataset contained 68 cells with 75% EXC, 15% NFSI and 10 FSI. These test datasets are used to determine the cross-species transfer of the performance (see **Fig. 3** and **SFig. 4**).
The UMAP embedding is based on all non-test data from all species and serves as a crucial step in integrating and aligning class-specific electrophysiological profiles by projecting the intrinsic membrane properties of all species into a shared, abstract space. To evaluate the test sample prediction performance, cells are projected into the UMAP coordinate space using the transform function obtained from the initial embedding. Prediction performance is evaluated by species/dataset (see Fig. 3B and SFig. 4).

10) It is not clear how certain aspects of the clustering/classifier were implemented. For instance, in the statement “Cell type composition of training data was informed by histological data of the primate neocortex, consequently the ratio of SST, VIP and NGF cells within the training data was fixed to 2:2:1.”; what is the reference for this? Classic studies have shown that the proportion of distinct interneuron types differ across areas, specifically along the visual occipito-temporal axis (Hendry et al. 1989; Kondo et al. 1994; Glezer et al. 1998; DeFelipe et al. 1999) in macaque monkeys.

We apologize for glossing over our reasoning for the composition of subpopulations within the (previous) C2 class (now NFSI). We acknowledge the classic studies cited and agree that the proportion of distinct interneuron types can differ across areas. However, for our model, we relied on transcriptomics/in-situ hybridization data rather than immunohistochemistry, because the latter did not provide a clear correspondence with our chosen labels (SST/VIP/NGF) that are established for the mouse.
To be more precise, our decision was based on a combination of Krienen et al. (2020) for the selection of the appropriate markers and the marmoset gene atlas (Kita et al., 2021). We examined cell densities in the upper layers of the LPFC (A8C, A6V) and found that SST density appeared highest but were comparable to that of VIP cells. Lamp5 densities (a marker for neurogliaform -NGF- cells), on the other hand, were qualitatively lower. We have not performed our own quantifications.
We also considered some mouse literature. For instance, Rudy et al. (2011) and Pfeffer et al. (2013) provide relative frequencies of major GABAergic subpopulations. Rudy et al. (2011) reported that in layers II/III of vibrissal somatosensory cortex, SST cells make up ~20% of GABAergic neurons, while 5-HT3A cells (which, among others, include VIP and NGF) make up ~50%. Pfeffer et al. (2013) reported for V1 in its entirety that SST-cre cells comprised 30% of GABAergic neurons, VIP cells 17%, and non-VIP 5-HT3A cells (which include NGFs) 16%. It is reasonable to assume that the proportions of VIP and SST cells would be more similar when only considering layers II/III of V1. Neurogliaform cells are found in all layers and have not been reported to show major density differences by layer (beyond layer I, in which they are most numerous).
In summary, we found that fixing the ratio of SST, VIP, and NGF cells to 2:2:1 was a reasonable and balanced solution that accounts for the general situation in many different species and brain areas, including the primate LPFC. We have added text to the Methods section to make our reasoning more transparent. Thank you for this valuable comment.

11) Similarly, for the statement: “Marmoset and human test data set for cross-species validation was determined by unambiguous morpho-electrophysiological identity”. – what do the authors mean by “unambiguous morpho-electrophysiological identity”?

Thank you for raising this important question. By "unambiguous morpho-electrophysiological identity," we mean that these cells were taken from a pool of candidates that exhibited a clear and consistent alignment between their morphological and electrophysiological properties, allowing determination of cell identity in the absence of genetic markers. For example, a cell was an unambiguous FSI cell if it had a characteristic basket cell morphology (e.g., non-pyramidal soma, multipolar dendritic tree and highly branched axon) and was a fast-spiking (FS) cell, which is the expected electrophysiological profile for the majority of parvalbumin-expressing basket cells (such as the FSIs shown in **Fig. 2C**). Similarly, NGF cells or vertically oriented bitufted cells that did not show a fast-spiking firing pattern, were considered NFSI cells (such as the cells shown in **Fig. 2C** last column from the left for both areas).

12) While experiments and data were acquired to be consistent with AIBS data, the authors noted an effect of acquisition system on AP waveforms (Figure S2). How does this compare to the AIBS data (what acquisition system did they use)? Also, even as it appears no clustering based on acquisition system was shown, it is important that the number of V1 and LPFC cells recorded in each system is balanced and is stated in the paper

We thank the reviewer for the opportunity to elaborate on this methodological concern. To our knowledge, the data from AIBS has been collected exclusively with a Multiclamp 700B amplifier, which was also most frequently used in recording our data set (43% of marmoset cells). Pipette capacitance neutralization in this system is done manually. It involves a reiterative calibration of several components (feedback resistor, bridge balance, capacitance neutralization), which requires some expertise. Consequently, just because waveforms were obtained from the same system does not mean they are automatically comparable. By default, there is no automatic documentation, but the AIBS has developed complementary software which we used in a majority of our MC 700B recordings. Hence, we can directly compare our compensation values (in pF) with the AIBS (see subsequent figure, blue boxplots). It is important to note that these values should not be interpreted as the actual amount of pipette capacitance.

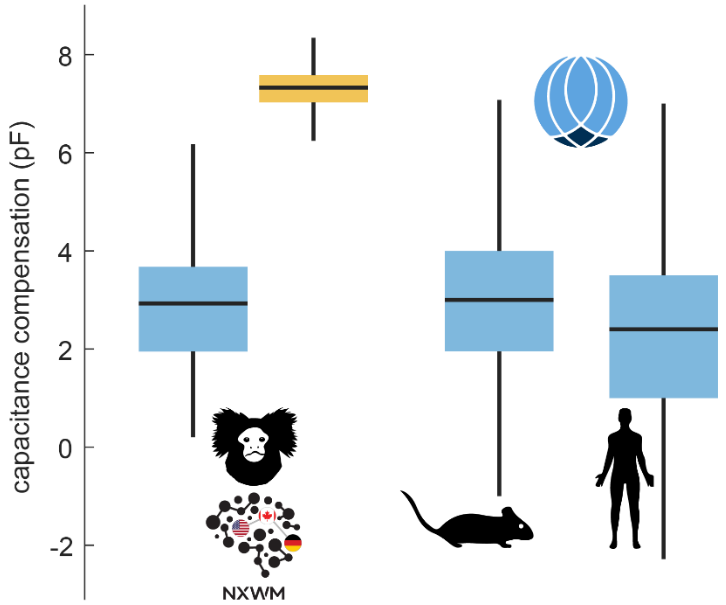

Capacitance compensation values in the HEKA EPC 10 (which is used for 27% of marmoset cells, golden color) are documented automatically. This compensation is based on measurements made in voltage clamp in on-cell configuration and are a more accurate reflection of the actual pipette capacitance. For cells recorded with the npi SEC system (30% of cells) we unfortunately do not have comparable documentation, because these circuits are controlled in a completely analog manner. Our documented capacitance neutralization from the same recording system is similar to recordings from the AIBS, but compensation values using the HEKA EPC 10 are much higher, which is in line with the observed effects on recorded waveforms (i.e. faster up and down stroke as well as higher AP amplitude in aspiny cells). Comparisons of capacitance compensation values across systems should be taken with a grain of salt, because of different electronic hardware. HEKA, for example, also has a variable time constant for capacitance compensation. We conclude from our study that the HEKA EPC 10 has a more potent compensation compared to the Multiclamp 700B. However, this may only matter for cells with rather fast action potentials, such as PV/FSI cells.

We give a detailed account of recording system effects by area in **SFig 2**. We are confident, that recording system was not a decisive influence on comparisons across areas despite showing a prominent effect on high-frequency AP features (particularly in FSI), such as peak slope and AP amplitude. Features that we excluded as inputs for classification out of caution, such as AP amplitude, did not show a significant area difference. In addition, we used a linear mixed model with experimenter and amplifier as nested random effects to verify that the effect of brain area on AP half-width was independent of such an influence. Modelling non-independence by amplifier and experimenter resulted in a better fit (BIC: -114.63 vs. -128.56, linear vs. mixed) but had very similar results for significance and effect size of the predictors brain area and membrane capacitance (see **STab. 4** and **SFig 2**).

13) Why was there no comparison of C2 type (non-fast spiking interneurons) between areas?

In the current version of the manuscript we show these as the group of NFSI interneurons and document the results. Differences between LPFC and V1 in NFSI interneurons were smaller, with a low number of significant effects and small effect sizes. Our resubmission features more visualizations and analysis of that group.

14) The abbreviation “hero” is not defined.

We apologize for insufficiently explaining what is meant by “hero (sweep)”. Some firing pattern quantifications/features are based on a singular sweep. The hero sweep is defined (adopted from the AIBS protocols) as the sweep closest to 65% of the sweep with max. firing rate. See supplementary table 2 for a description.

Reviewer 2

General Comment

The current study asks if differences in hierarchical visual areas arise from their connectivity using roughly similar cell types or from intrinsic neuron properties. They use marmoset monkeys to examine two opposite ends of primate visual system, V1 and LPFC, and measure intrinsic membrane properties of patch clamped neurons plus fill them to get morphology. In electrophysiology it is well appreciated that representations in V1 are more stable in their feature preferences over time whereas LPFC is much more plastic and involved in working memory. Thus, the possibility that some of these differences in function could be supported by differences in neuron properties is an exciting question. By contrast there is not much support for such difference between neuron properties in mice (which have been more extensively studied using these methods), and if primates do exhibit strong differences it could underlie an important part of species difference and the greater cognitive flexibility achieved in primate behavior. The work presented is important and the methods applied and resulting data are valuable and of high quality.

The results of this study first establish a basic set of cell types (three broad classes, fast spiking, non-fast spiking non-spiny neurons, and excitatory spiny neurons) and show how they compare to former studies in mice and humans. Then they examine differences between V1 and LPFC in the marmoset and find several significant and interesting differences: 1) LPFC excitatory neurons have a higher threshold to fire (correspondingly lower mean rates), 2) LPRC fast spiking cells has longer latencies to fire, 3) LPFC excitatory and fast spiking neurons were more likely to have burst firing. These intrinsic firing differences could support some differences in function between V1 and LPFC in regards to plasticity of representations and working memory. Overall I found the manuscript well written with clear illustrations of data.

We thank the reviewer for the positive view of the manuscript and for the detailed comments below. In the current version, we have considerably expanded our sample size and data analyses, and we place more emphasis on the differences we found in the e-features of inhibitory interneurons. Below, are our detailed answers to the specific comments.

1) Marmoset/Human data cluster different than mouse

On Fig 2C, A) the marmoset/human cells seem to cluster in different parts of the space than the total UMAP clusters which mainly are driven by mouse data). Is this difference significant? This might just be that there are so many mouse (gray points in Fig 2C upper left) that it appears like a uniform covered area whereas the density in mice is actually comparable to that in human/marmoset showing more sampling in the central region of the main UMAP cluster rather than the edges. What would be the implications if this is a real distribution difference (less diversity in marmoset and human types?).

We appreciate the reviewer's observation regarding the uneven distribution of primate cells in now Figure 2 and agree that a considerable amount of marmoset cells (and human to a lesser extent) appear to occupy different regions of the UMAP space. There are several reasons why this is the case:
First, the UMAP projection is non-linear, and interpretation of densities and distances (in 2D-visualization) is thus not straightforward. Second, neuronal sampling during recordings is quite distinct in our study relative to the mouse data from the AIBS, where cell types were mapped using Cre-reporter systems. Marmoset and human cells are selected by the experimenter using morphological criteria (e.g., soma shape). Fluorescent targeting (using Cre-reporters) of distinct cell types in mice reduces experimenter bias toward a particular cell shape or size. Similarly, mouse cells were sampled across all layers, whereas marmoset neurons are primarily from supragranular layers I-III. Third, sample size is an important factor when representing data in UMAP space. There are more mouse data points than marmoset data points. However, marmoset cells often have high input resistance and sag, which may lead to being pooled close together. Interestingly, our updated analysis, which includes 109 additional marmoset neurons, also appears to yield larger marmoset clusters with greater segregation from the mouse clusters, but presumably also higher differentiation within them. Interestingly, the classifier trained on mouse data still segregates the three main categories we use in our study (EXC, NFSI, and FSI), indicating that some of the core features of these cell types are preserved across species. We need to explore this issue in more detail in a separate line of work using viral vectors with fluorescent markers to segregate different cell types in marmosets. This work is now starting. In the present paper, we focus on inter-area differences among the three aforementioned main cell classes in marmosets. We use UMAP visualization as a screening tool to categorize the three main neuron classes that we explore in our study. These broad categories seem to generalize across species.

2) Significance and effect of the SAG ratio

For the SAG difference mentioned in text, is the difference statistically significant, and how was it tested (stat not included though the difference looks clearly significant visually). Can more be said briefly about how a higher SAG effects neuron firing … it would lead to lower or higher firing rates, to more or less spike rate adaptation?

We thank the reviewer for this comment. With the data we have added, we have made interesting observations about sag. We compared sag between V1 and LPFC neurons, and across all neuronal groups, sag was larger in V1 (see the last paragraph of the results section, **"**Features of e-types in areas V1 and LPFC"). Sag can be linked to properties such as input resistance, which, although it seems in general higher in V1 than in LPFC, shows a diverse pattern that depends on cell type (See **Fig. 4A** and Discussion section "Differences in intrinsic properties and morphology between cell types in V1 and LPFC"). Sag can also be related to membrane time constant; increased sag dynamically shortens the effective membrane time constant without necessarily changing its resting value. However, these relationships could be complex, and sag, input resistance and membrane time constant can vary independently. To clarify this issue, one should quantify HCN channel expression across different cell types and disentangle the contribution of these channels to the different properties. We did not do that in the present study, therefore we can reach only limited conclusions. We now state so in the Discussion section.

Regarding the interspecies comparison of sag, which we assume the reviewers refers to, we performed a 2-way ANOVA with species and cell type as factors to examine the significance of the difference between mouse and marmoset V1 cells (see **STab. 5**). Sag ratio difference was clearly significant ($p < 0.0001$), with a sizable species effect (partial eta-squared = 0.192). However, we interpret these results with caution. This comparison needs to be restricted to similar areas, layers, and cell types. Because the mouse data were restricted to V1, this comparison must focus on this area. We are working on a separate study that addresses sag and includes quantification of HCN channels. We focus on the inter-area comparison in the marmoset for the current study and state the differences between areas and possible relationships to other variables (see Discussion section "Differences in intrinsic properties and morphology between cell types in V1 and LPFC").

3) Training data for the classifier

It should be stated more clearly what inputs were used to train the classifier (presumably the ephys data from Fig 2C, AP width, latency, SAG ratio?) but not morphology data fom Fig 2D?). This could go in the first paragraph of this section where forest classification is first described. What is shown in Figure 2E is also unclear and needs better explanation.

We thank the reviewer for pointing this out. The new version of our manuscript explicitly states, on several occasions, that the UMAP projection was based solely on intrinsic e-features derived from our whole-cell patch-clamp recordings. These features include, but are not limited to, those visualized in Figure 2B. We agree it is important to clarify that morphological data were not used as an input for the UMAP embedding. In the revised manuscript, we ensure that the text clearly articulates these points (e.g., Results subsection "Classification of Marmoset Cell Types Based on Intrinsic Properties", first paragraph).

4) Clarification of firing rate impact for Ri, Rheo and Rect; Term definitions

Could the authors provide a brief sentence to describe the impact in terms of firing in neurons for input resistance, rheobase, and rectification? It is only mentioned in the text that this would mean LPFC cells are less excitable, but I suspect this would also change their membrane time constants for integration (i.e., are LPFC cells slower and more sluggish in their firing, slower time constants). Also the text mentions spike rate adaptation as larger in LPFC … does that derive from these parameters, perhaps from the higher rheobase (threshold for action potential generation). What is the "herosweep" and how would it affect firing? Some brief definition of terms would be useful for a more general audience.

We thank the reviewer for this comment and the opportunity to clarify these issues. Generally, higher input resistance reflects passive membrane resistance and is associated with higher firing rates at a fixed stimulation level. Similarly, cells with higher input resistance in-vitro should show higher firing rates in-vivo (see for example pyramidal cells in V1 vs. LPFC). Longer time constant could lead to further neuronal firing for prolonged stimuli or at the occurrence of subsequent input/stimuli in shorter intervals. It is a challenge to consider these variables in isolation. The time constant of the cell membrane usually increases in proportion to the input resistance.
Several quantifications of a neuron's spike train are taken from a singular sweep, hence the name "hero sweep". It is defined as the sweep closest to 65% of the sweep with maximal firing rate. Consequently, the current injected during the hero sweep is not fixed (such as + 80 pA over rheobase). See S Table 2 for definitions.

5) Presentation of Fig. 6 and missing STD

Results for Fig 6 seems clear, only wish to change the shading in 6C and 6D to better show when the differences are significant (maybe use 2 std for the shaded areas rather than full range?). Also it should be better indicated which of those differences are significant.

We have changed the format of this figure, and now the differences are more apparent.

6) In discussion, paragraph 1, it is unclear what this phrase, "a more depolarized action potential trough" means and seems to introduce a result that I must have missed in the results section … perhaps this finding should be emphasized better in the text of results. I take it that a different action potential shape (less negative deflection in the trough) was seen for the fast-spiking C1 cells in LPFC than those in V1 … are there any implications for their function that they have this property?

We apologize for our lack of clarity. In the current version of the manuscript, we have replaced the description of the three main classes, C1, C2 and C3 by a generic naming/description of the main cell types that can be related to known electrophysiological categories, ECX, NFSI, and FSI neurons. We also clarify and discuss the differences in e-features between cell classes. A more depolarized AP trough refers to a smaller AHP amplitude due to sustained depolarization. For FSI with a high burst index, a more depolarized AP potential may mean that the neurons can produce successive spikes separated by short ISIs, favoring burstiness. However, this issue must be investigated in detail using a larger sample of bursting FSI and other tests to establish whether different conductances may contribute to this effect. In the current version of the manuscript, we refer to the burst index and possible factors contributing to it in the Discussion (see "Bursting as a distinctive feature of LPFC neurons subsection").

7) In Figure 4F, the difference seem to be circularly defined? I suppose that one thing to clarify is that burst or not-burst is defined by an intra-cellular test to evoke a burst, but then the burst index I assume is defined using a different index from firing. Can that be clarified?

We apologize for not making clear how the burst index was calculated. We realize that defining intrinsic bursters is a complicated issue and we have not done enough tests to label neurons as intrinsic bursters. We now decided to use one burst index and define cells along a continuum. The results are shown in Fig. 5 and the definition of burst index appears in the Methods section (last sentences of "Analysis of electrophysiological features" subsection).

Reviewer 3

General Comment

The manuscript "Single neuron diversity supports area functional specialization along the visual cortical pathways" by Feyerabend et al. is a multi-center effort that characterizes the morphological and electrophysiological properties of >140 single cells in marmoset V1 and prefrontal cortex (PFC) and compared them to mouse and human cell type properties. The study quantifies that PFC neurons are larger, less excitable and fired broader spikes an V1 cells, that subgroups of marmoset interneurons have long axons and slower response latencies, and that burst firing was more common in PFC excitatory and inhibitory cells. These area- and species- specific findings are described in the context of an impressive overall commonality of the distinct features of fast spiking versus spiny principal cells.

The study reaches these conclusions with state-of-art techniques, a comprehensive cell characterization protocol, advanced clustering approaches and fair statistical evaluations. The writing is concise, and the conclusion are well justified.

Overall, this is an impressive study that provides an important characterization of cell types in visual and prefrontal cortex of marmosets, clarifying their differences to cells types characterized in the mouse and discussing apparent unique characteristics of some cell types.

The study has the potential to become a key reference for future studies aiming at understanding cell type specific functions in V1 and LPFC of the marmoset. The results are critical for interpreting the functional specializations of the visual and prefrontal cortex in primate brains. The provided results may prove important for understanding cell type specific alterations in psychiatric 'prefrontal cortex' disorders.

We appreciate the reviewer's optimistic view of our manuscript and its contribution to the understanding of cell type diversity across different brain areas, as well as the comments that have helped improve the manuscript. Our answers are below.

1) It is not clear why the characterization of the interneuron types assigned to class 2 is missing. Readers are exposed to neuron types that are assigned to class 2, but do not receive information on how variable the class 2 cells are. This information is missing but would help a reader to discern how different (on average) C2 class neuron properties are to the C1 and C3 class. The authors may consider a supplementary results section that briefly describe what the e-types 1, 2-3, and 4 are that characterize class 2 neuron types, and add a table about electrophysiological or other properties of cells in that class.

We thank the reviewer for this comment, which has motivated us to substantially revise this aspect of the manuscript. First, we have replaced the descriptions of classes C1, C2, and C3 with three categories of cell types, consistent with the current literature (EXC, NFSI, and FSI neurons). We now expand on the analyses of C2 neurons (NFSI interneurons in the current version). We found very few differences between LPFC and V1 NFSI interneurons e-features. It is plausible to us that many of these cells, such as NGF (or L1) cells, show less divergence between areas. Still, we are hesitant to draw a firm conclusion because we cannot presently account for finer differences in cell-type composition. Some NFSI cells, however, show very distinct characteristics such as high input resistance and sag ratio as well as intermediate spike width and firing rates. The new version of the manuscript features more visualizations and analyses of this group (see **Figs 4** and **5, Table 1** and **Supplementary Table 1**) and we refer to our findings in the Discussion section (various subsections).

2) Classification of the cell group labels (C1,2,3) used the UMAP components (the results text says so) or a subset of PCAs of the raw features (the methods describe this). It is not made clear why these decisions were made, as the classification would be closer to the actual cell properties if the classifier used the morphological and electrophysiological features instead of the UMAP / PCA components. These components are already describing the most distinguishable features so that a classifier may have high label assignment accuracies because the input already pre-clustered them.

A related question is why the authors decided on using a 2 and 3 UMAP component visualization instead of performing unsupervised clustering of the data and testing how consistent marmoset cell labels are assigned to the clustered classes. It is not apparent what the advantage is - if any - of the chosen approach.

It would enhance the appeal of the study if a clustering and cluster visualization approach is used that allows a direct comparison to the relevant literature of cell types (in mice or monkeys), or explicitly discuss the rationale for choosing the UMAP approach (mostly for visualization?).

Thank you for your thoughtful and constructive comments regarding our use of UMAP and supervised classification. We appreciate the opportunity to clarify our methodological choices. Our decision to use UMAP features was strategic, aimed at creating a more 'generalizable' classifier that leverages existing information about labels (mouse) and dendritic type (marmoset and human), while reducing the dimensionality of the feature space and enabling visualization. However, we repeated the analyses using all e-features as inputs and obtained the same results. Our feature set includes 29 properties, many of which are correlated (e.g., input resistance and rheobase) and are susceptible to noise in single-sweep measurements. UMAP is a powerful dimensionality reduction technique that mitigates such issues by preserving the underlying manifold structure of the data, thereby eliminating unsystematic variation and redundancy. This allows the classifier to operate on a more compact representation.
We also want to emphasize that UMAP was a vital tool for visualization and guidance. We believe UMAP is an accessible way to convey the diversity of our dataset to the broader community (see e.g., Lee et al., 2023), which is particularly important as we prepare to make the data publicly available.

In response to the reviewer's suggestion, we have now performed a direct comparison between our UMAP-based approach and a purely e-feature-based (Fig. 3 and SFig. 4) approach. The results are consistent with our rationale: the e-feature-based classifier achieved better test performance on mouse data, while the UMAP-based approach more accurately classified test datasets from marmosets. However, the performance differences were marginal. We additionally decided to use the UMAP classifier because it aligns the results with the visualizations shown in the Figures.

The reviewer raises a point about comparing our findings with unsupervised clustering results. Gouwens et al., 2018, showed that e-types were clustered by dendritic type. Furthermore, they found 5 different clusters for FSI cells, for example, but none of them seem to correspond to a certain layer or morphological type, such as chandelier cells. The outcome of unsupervised clustering may be strongly influenced by factors such as sample size, sampling strategy, features used, etc. Our goal, on the other hand, was to apply a consistent taxonomy across the main cell types and compare areas V1 and LPFC. That said, as the reviewer pointed out, unsupervised classification might be an excellent exploratory technique, as used in Krimer et al. 2005, to illuminate the relationship between morphological types and e-phenotypes. Unfortunately, even though our dataset is large relative to previous e-studies in non-human primates, our sample size (particularly for morphological labels) is insufficient for this approach. We are expanding our samples and refining our anatomical reconstructions so we can follow that advice in the future.

3) Why does Figure 6 show the range but not the standard deviation or standard error of the morphological features?

We thank the reviewer for this comment. We originally showed the range to better reflect the morphological variety. But we agree with the reviewer that the standard deviation is a better choice and changed the figures accordingly. This figure has also changed after we added additional reconstructions.

4) p. 7 what are the "e-type[s] ranging from Inh_4 to Inh_7" and what are "e-type 2 or 3 " ? Are there e types that uniquely characterize SST and VIP neurons? How similar are the distributions of these e-Types in the marmoset and mouse datasets?

We thank the reviewer for pointing this out. We borrowed these labels from Gouwens et al. 2018., based on semi-supervised clustering of electrophysiological data obtained from mouse. We changed the wording calling them “AIBS e-types" in the manuscript to make this clearer. Generally, there is an association between electrophysiological “phenotype” and cell identity. So, we include AIBS e-type as an additional criterion to ensure non-overlapping ground truth labels. We attribute each e-type to a specific subpopulation of neurons (see SFig 1). We associate SST cells with e-types 4-7 and VIP cells with e-types 2-3. For a detailed breakdown of e-types and transgenic lines please review SFig 12 of the aforementioned publication. Below are some examples of e-features by AIBS e-type for inhibitory neurons. The color code follows the UMAP colors in Fig. 2 and SFig. 1.

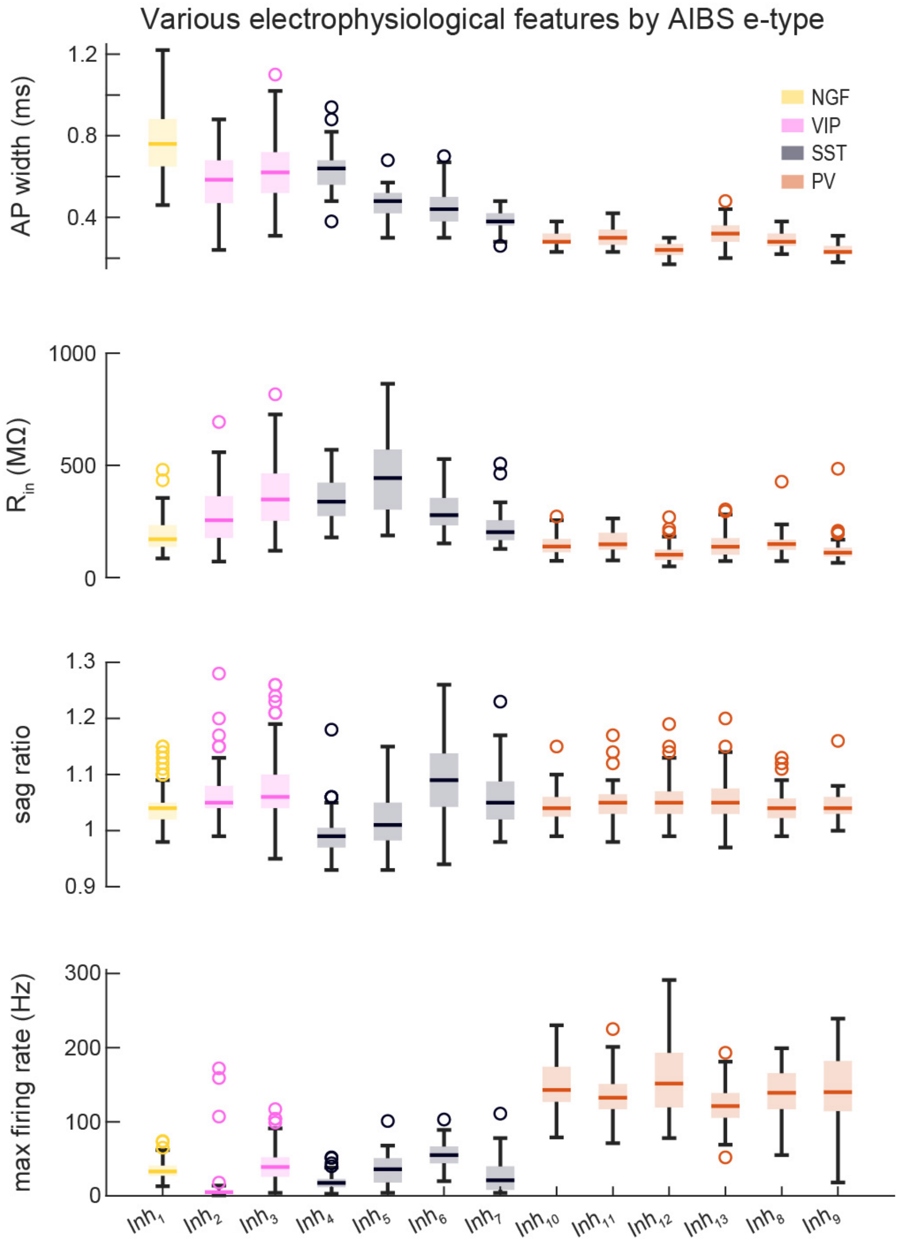

Supplement: Supplementary file 3 — Transparent Peer Review file [file 41467_2026_73734_MOESM3_ESM.pdf]
